# Periostin Directly and Indirectly Promotes Tumor Lymphangiogenesis of Head and Neck Cancer

**DOI:** 10.1371/journal.pone.0044488

**Published:** 2012-08-30

**Authors:** Yasusei Kudo, Shinji Iizuka, Maki Yoshida, Phuong Thao Nguyen, Samadarani B. S. M. Siriwardena, Takaaki Tsunematsu, Mariko Ohbayashi, Toshinori Ando, Daijiro Hatakeyama, Toshiyuki Shibata, Keiichi Koizumi, Masahiro Maeda, Naozumi Ishimaru, Ikuko Ogawa, Takashi Takata

**Affiliations:** 1 Department of Oral and Maxillofacial Pathobiology, Graduate School of Biomedical Sciences, Hiroshima University, Hiroshima, Japan; 2 Department of Oral and Maxillofacial Sciences, Gifu University Graduate School of Medicine, Gifu, Japan; 3 Department of Kampo Diagnostic, Institute of Natural Medicine, University of Toyama, Toyama, Japan; 4 Department of Research and Development, Immuno-Biological Laboratories, Co., Ltd., Fujioka-shi, Gunma, Japan; 5 Center of Oral Clinical Examination, Hiroshima University Hospital, Hiroshima, Japan; 6 Department of Oral Molecular Pathology, Institute of Health Biosciences, The University of Tokushima Graduate School, Tokushima, Japan; Ospedale Pediatrico Bambino Gesu', Italy

## Abstract

**Background:**

Metastasis to regional lymph nodes via lymphatic vessels plays a key role in cancer progression. Tumor lymphangiogenesis is known to promote lymphatic metastasis, and vascular endothelial growth factor C (VEGF-C) is a critical activator of tumor lymphangiogenesis during the process of metastasis. We previously identified periostin as an invasion- and angiogenesis-promoting factor in head and neck squamous cell carcinoma (HNSCC). In this study, we discovered a novel role for periostin in tumor lymphangiogenesis.

**Methods and Findings:**

Periostin overexpression upregulated VEGF-C mRNA expression in HNSCC cells. By using conditioned media from periostin-overexpressing HNSCC cells, we examined tube formation of lymphatic endothelial cells. Conditioned media from periostin-overexpressing cells promoted tube formation. To know the correlation between periostin and VEGF-C, we compared Periostin expression with VEGF-C expression in 54 HNSCC cases by immunohistochemistry. Periostin expression was correlated well with VEGF-C expression in HNSCC cases. Moreover, correlation between periostin and VEGF-C secretion was observed in serum from HNSCC patients. Interestingly, periostin itself promoted tube formation of lymphatic endothelial cells independently of VEGF-C. Periostin-promoted lymphangiogenesis was mediated by Src and Akt activity. Indeed possible correlation between periostin and lymphatic status in periostin-overexpressing xenograft tumors and HNSCC cases was observed.

**Conclusions:**

Our findings suggest that periostin itself as well as periostin-induced upregulation of VEGF-C may promote lymphangiogenesis. We suggest that periostin may be a marker for prediction of malignant behaviors in HNSCC and a potential target for future therapeutic intervention to obstruct tumoral lymphatic invasion and lymphangiogenesis in HNSCC patients.

## Introduction

Millions of people die each year of metastatic cancer. Metastasis occurs via the blood or lymphatic vessels or directly into tissues and body cavities. Although the biochemical mechanisms of metastasis are poorly understood, the process is thought to be systematic rather than random [1]. Regional lymph nodes are frequently the first sites of spread, presumably due to tumor cell drainage via pre-existing afferent lymphatic vessels and/or newly formed lymphatic capillaries [2], [3]. Head and neck squamous cell carcinoma (HNSCC) is one of the most common types of human cancer, with an annual incidence of over 500,000 cases worldwide [4]. The literature suggests that the most important reason for the high mortality rate is that the disease is often not diagnosed or treated until it has reached an advanced stage. Despite aggressive, multidisciplinary treatment approaches, including preoperative or postoperative chemotherapy and/or radiotherapy with reconstructive surgery, 5-year survival of HNSCC has not improved significantly over the past 20 years [5]. Like most other epithelial cancers, HNSCC develops in a multistep process through the accumulation of multiple genetic and epigenetic alterations. The most important prognostic indicator for HNSCC patients is metastasis to the cervical lymph nodes or distant organs [6]. We previously established an HNSCC cell line from a metastatic lymph node [7] and used an *in vitro* invasion assay to isolate a highly invasive clone from this cell line [8]. We then compared the transcriptional profiles of parent HNSCC cells and the highly invasive clone by microarray analysis and identified periostin (osteoblast-specific factor 2) as the gene most differentially expressed in the invasive clone [9]. Periostin is a secreted protein that has been suggested to function as a cell adhesion molecule for pre-osteoblasts and to participate in osteoblast recruitment, attachment, and spreading [10], [11]. Periostin overexpression enhanced invasion and anchorage-independent growth in HNSCC cells [9]. Interestingly, periostin-overexpressing cells were aggressively invasive and spontaneously metastasized to cervical lymph nodes and to the lung in an orthotopic mouse model of HNSCC [9]. Bao et al. also demonstrated that a colon cancer cell line with low metastatic potential displayed strikingly accelerated tumor metastatic growth in an animal xenograft model of metastasis when engineered to overexpress periostin [12]. These findings indicate that periostin overexpression may be common in various types of cancer and that periostin may be important for tumor invasion. Periostin has previously been shown to stimulate metastatic growth by inducing angiogenesis [12], [13] and enhances VEGF receptor Flk-1/KDR expression in endothelial cells through an integrin αvβ3-FAK-mediated signaling pathway [13]; furthermore, recombinant periostin enhances capillary formation *in vitro* [14]. Importantly, clinical studies of periostin expression in human cancers have demonstrated that increased expression of periostin correlates with the number of tumor blood vessels and with metastasis [14]. On the other hand, previous immunohistochemical studies showed a possible correlation between periostin expression and lymph node metastasis in cancer cases [15]. However, there is no study on the role of periostin in tumor lymphangiogenesis. In the present study, we demonstrate a novel action of periostin: the direct and indirect promotion of tumor lymphangiogenesis.

## Results

### VEGF-C upregulated by periostin overexpression promotes tube formation of lymphatic endothelial cells

**Figure 1:**
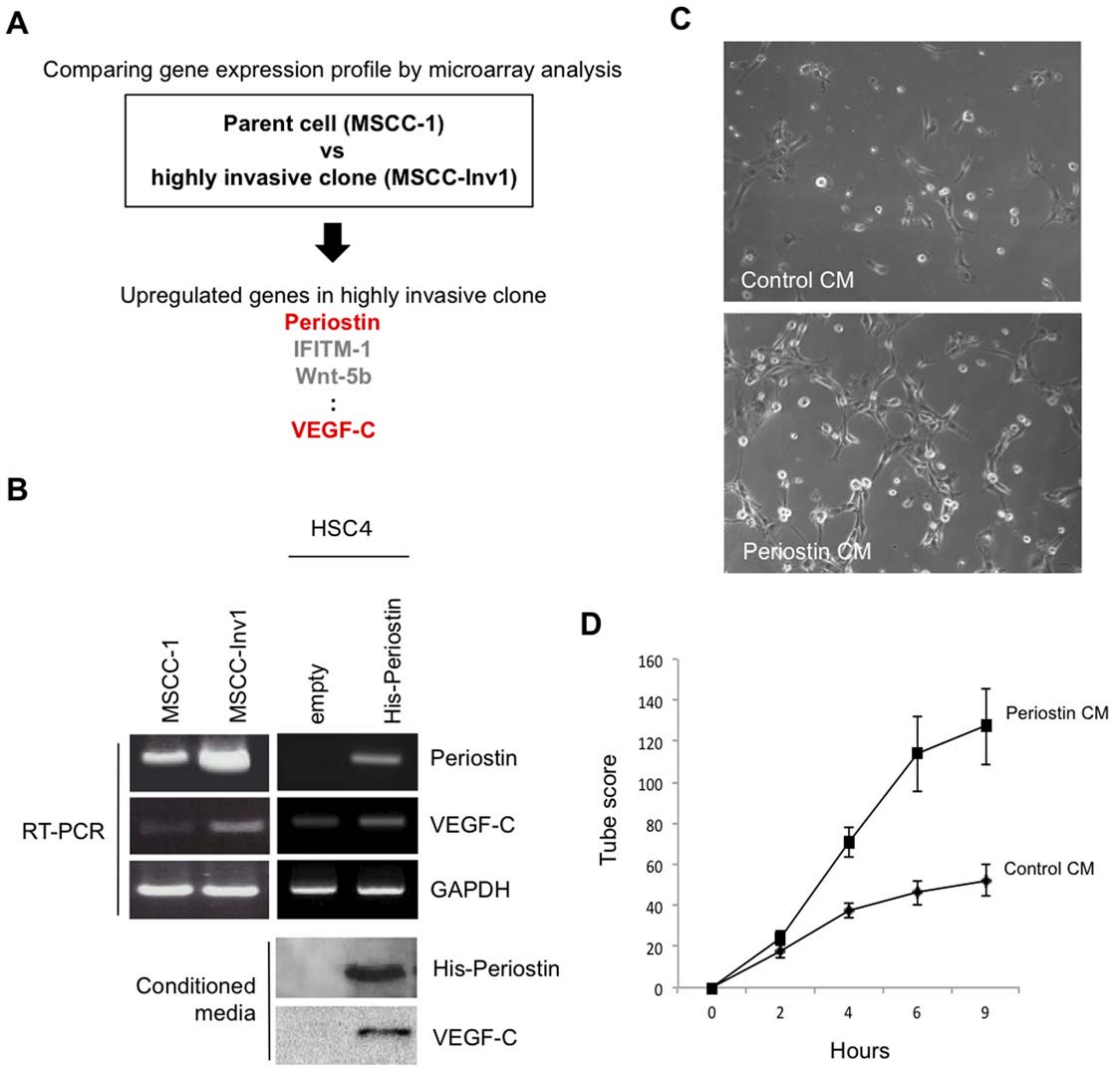
Involvement of VEGF-C promoted by Periostin overexpression on the tube formation of TR-LE cells. (A) Schema shows the strategy to identify Periostin and VEGF-C by comparing the gene expression profile between the parent (MSCC-1 cells) and a highly invasive clone (MSCC-Inv1 cells). (B) Higher expression of VEGF-C mRNA in cells of the highly invasive clone MSCC-Inv1 and VEGF-C expression in periostin-overexpressing cells. HSC4 cells without periostin expression were transduced using a retroviral plasmid encoding hexa-histidine-tagged periostin. Periostin and VEGF-C mRNA expression levels in MSCC-1 cells, MSCC-Inv1 cells, empty vector-transfected HSC4 cells (empty), and periostin-overexpressing HSC4 cells (His-periostin) were examined by RT-PCR. GAPDH expression was used as a loading control. Conditioned media were collected from empty vector-transfected HSC4 cells (empty) and periostin-overexpressing HSC4 cells (His-periostin) after incubation for 4 days. Conditioned media were concentrated and analysed by western blotting for expression of His-periostin and VEGF-C in conditioned media. (C) Conditioned media from periostin-overexpressing cells promotes tube formation of lymphatic endothelial cells. Tube formation of TR-LE cells by adding conditioned media from periostin-overexpressing cells. TR-LE cells were seeded onto matrigel-coated wells in the presence of conditioned media from empty vector-transfected (control CM) or periostin-overexpressing (periostin CM) HSC4 cells. After incubation for 0–9 h, the lengths of the tube-like structures formed were evaluated. The figure shows the cells after incubation for 9 h. (D) The graph shows the tube scores after 0–9 h incubation of control CM or periostin CM. The bars show the average values and SDs from 3 independent experiments.

We previously identified periostin as an invasion-promoting factor by comparing the gene expression profiles of the parent HNSCC cells (MSCC-1) and a highly invasive clone (MSCC-Inv1) [9] (Figure 1A). VEGF-C was also upregulated in the highly invasive clone by microarray analysis (Figure 1A). Increased VEGF-C expression in MSCC-Inv1 cells relative to parent cells was observed (Figure 1B). Moreover, in our previous microarray analysis to compare the gene expression profile between control and periostin-overexpressing HNSCC cells, VEGF-C was upregulated in periostin-overexpressing cells [9]. Here we confirmed that ectopic overexpression of periostin upregulated VEGF-C expression (Figure 1B). Both periostin and VEGF-C were detected in conditioned media from periostin-overexpressing cells but not control cells (Figure 1B). VEGF-C is known to be a critical activator of tumor lymphangiogenesis during the process of metastasis. Therefore, we examined tube formation of lymphatic endothelial cell by adding conditioned media from empty vector-transfected cells or periostin-overexpressing cells to TR-LE cells. TR-LE cells were previously established as an immortalized rat lymphatic endothelial cell line [16]. Conditioned media from periostin-overexpressing cells remarkably promoted tube formation by TR-LE cells (Figure 1C and 1D).

To demonstrate the effect of the periostin-induced VEGF-C expression on tube formation, we used the VEGF receptor 3 (VEGFR-3) kinase inhibitor MAZ51, which is reported to block both VEGF-C- and VEGF-D-induced phosphorylation of VEGFR-3 in PAE cells [17]. TR-LE cells is known to express VEGFR-3 [16]. We confirmed that MAZ51 markedly inhibited VEGF-C-induced tube formation (Figure S2A and S2B). MAZ51 greatly inhibited tube formation induced by conditioned medium from periostin-overexpressing cells (Figure S2A and S2B). The inhibition of tube formation by MAZ51 in conditioned medium from empty vector-transfected cells was also observed. This inhibition may be accounted for by VEGF-C secretion from lymphatic endothelial cells themselves. The inhibitory effect of MAZ51 was low in cells treated with conditioned medium from empty vector-transfected cells (Figure S2C). These findings indicate that periostin may promote lymphangiogenesis through upregulation of VEGF-C.

### Correlation of periostin expression with VEGF-C and lymphatic status in clinical cancer cases

**Figure 2:**
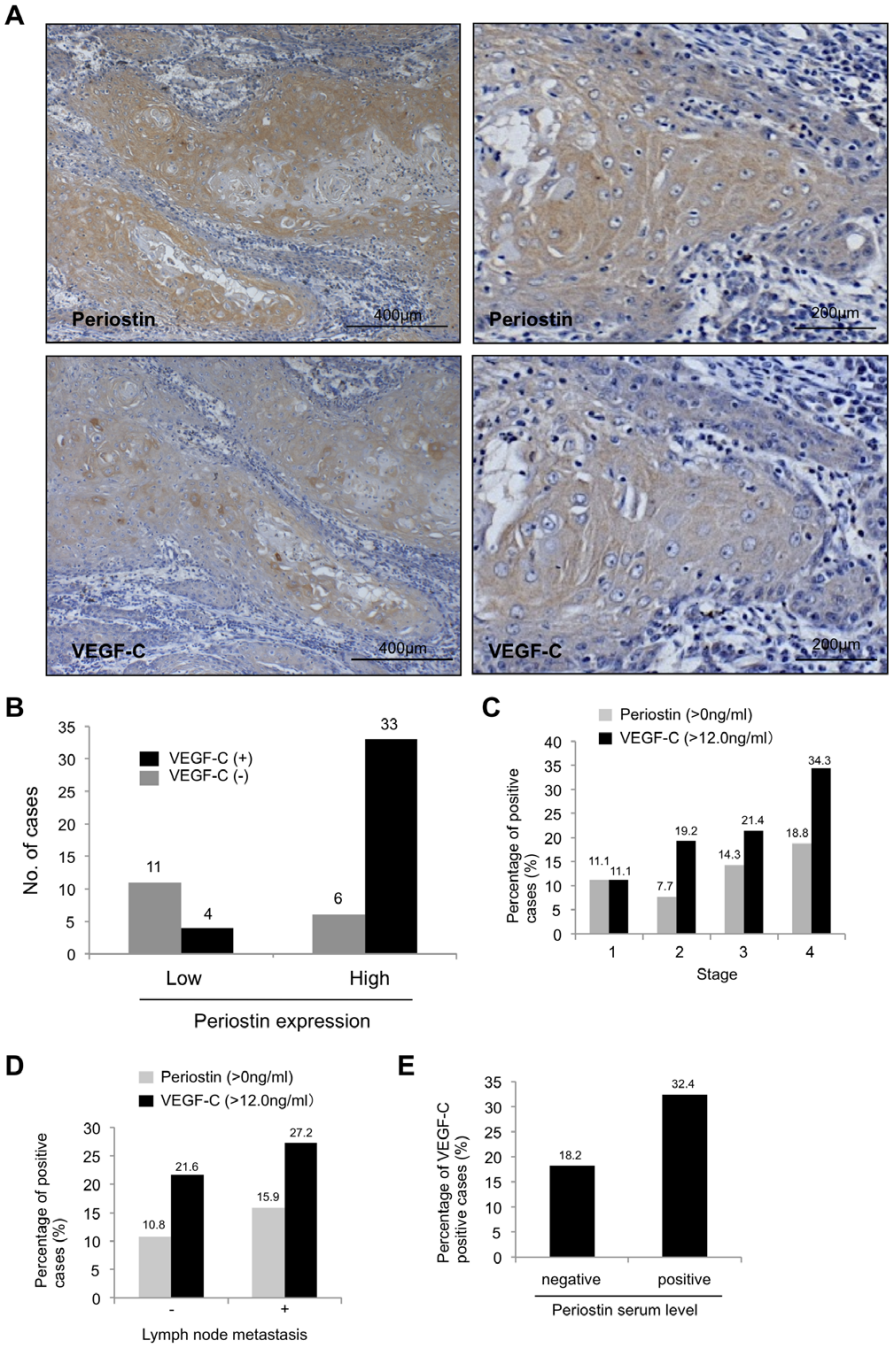
Correlation between periostin and VEGF-C in HNSCC. (A) Immunohistochemical staining for periostin and VEGF-C in HNSCC cases. Representative HNSCC cases (high and low magnification) with periostin and VEGF-C expression are shown. Scale bar is shown in each picture. (B) Graph shows the VEGF-C expression status in HNSCC cases with high or low expression of periostin. (C) Serum level of periostin and VEGF-C in 81 HNSCC patients was examined by ELISA. Serum level of periostin and VEGF-C was compared with tumor stage. Graph shows percentage of periostin or VEGF-C positive cases in different tumor stage (from stage 1 to 4). (D) Serum level of periostin and VEGF-C was compared with lymph node metastasis. Graph shows percentage of periostin or VEGF-C positive cases in cases with or without lymph node metastasis (E) Serum level of periostin was compared with serum level of VEGF-C. Graph shows percentage of VEGF-C positive cases in cases with periostin positive or negative.

To determine the correlation between periostin and VEGF-C expression levels in clinical cancer cases, we compared periostin expression with VEGF-C expression in 54 HNSCC cases by immunohistochemical analysis. We defined the grading of periostin and VEGF-C staining as high (over 10% of tumor cells showing ++ or +++ intensity) or low (no staining or less than 10% of tumor cells showing + intensity). For the criterium of 10% positive cells, we considered that HNSCC cases with less than 10% of tumor cells showing weak/focal immunopositivity was same as HNSCC case with no positive staining. Figure 2A shows a representative case of high expression of both periostin and VEGF-C. Both Periostin and VEGF-C expression were observed in cytoplasm of HNSCC cells (Figure 2A). Indeed, most cases with high expression of periostin or VEGF-C showed strong and diffuse immunopositivity as shown in Figure 2A and most cases with low expression of periostin or VEGF-C showed no immunopositivity as shown in Figure S3A. Only a few cases showed the heterogenous staining (Figure S3B). For the heterogenous staining, we totally evaluated the number of stained cells and their staining intensity by checking at least ten fields including superficial, central and deep invasive areas of the tumor. Of the 54 HNSCC cases, high expression of periostin was observed in 39 (72.2%) cases and high expression of VEGF-C in 37 (68.5%) cases (Figure 2B). Thirty-three of the 39 (84.6%) HNSCC cases with periostin expression expressed VEGF-C; this correlation was statistically significant (P<0.001).

As both periostin and VEGF-C are secreted proteins, we examined the serum levels of periostin and VEGF-C in HNSCC patients by ELISA. In this study, we used peripheral blood collected from 81 HNSCC cancer patients. These cases were different from cases used in immunohistochemical study. Clinical information including lymph node metastasis, tumor stage and TNM classification was gathered from surgical records of the patients (Table S2). In samples from 5 healthy controls, the periostin serum level was 0 ng/mL and the VEGF-C serum level less than 11.0 ng/mL (data not shown). Periostin was detected at 11.4 ng/mL in conditioned media from MSCC-Inv1 cells. Based on these results, a periostin-positive case was defined as having a serum periostin level >0 ng/mL and a VEGF-C-positive case as having a serum VEGF-C level ≥12.0 ng/mL. The percentage of periostin-positive cases increased with the stage of progression and with lymph node metastasis (Figure 2C and 2D). The data on serum levels of periostin and VEGF-C and the relevant clinical data on the HNSCC patients are listed in Table S2. The percentage of VEGF-C-positive cases also increased with the stage of progression and with lymph node metastasis (Figure 2C and 2D). Interestingly, 32.4% of periostin-positive cases but only 18.2% of periostin-negative cases were VEGF-C-positive (Figure 2E).

### Periostin directly promotes tube formation by lymphatic endothelial cells

**Figure 3:**
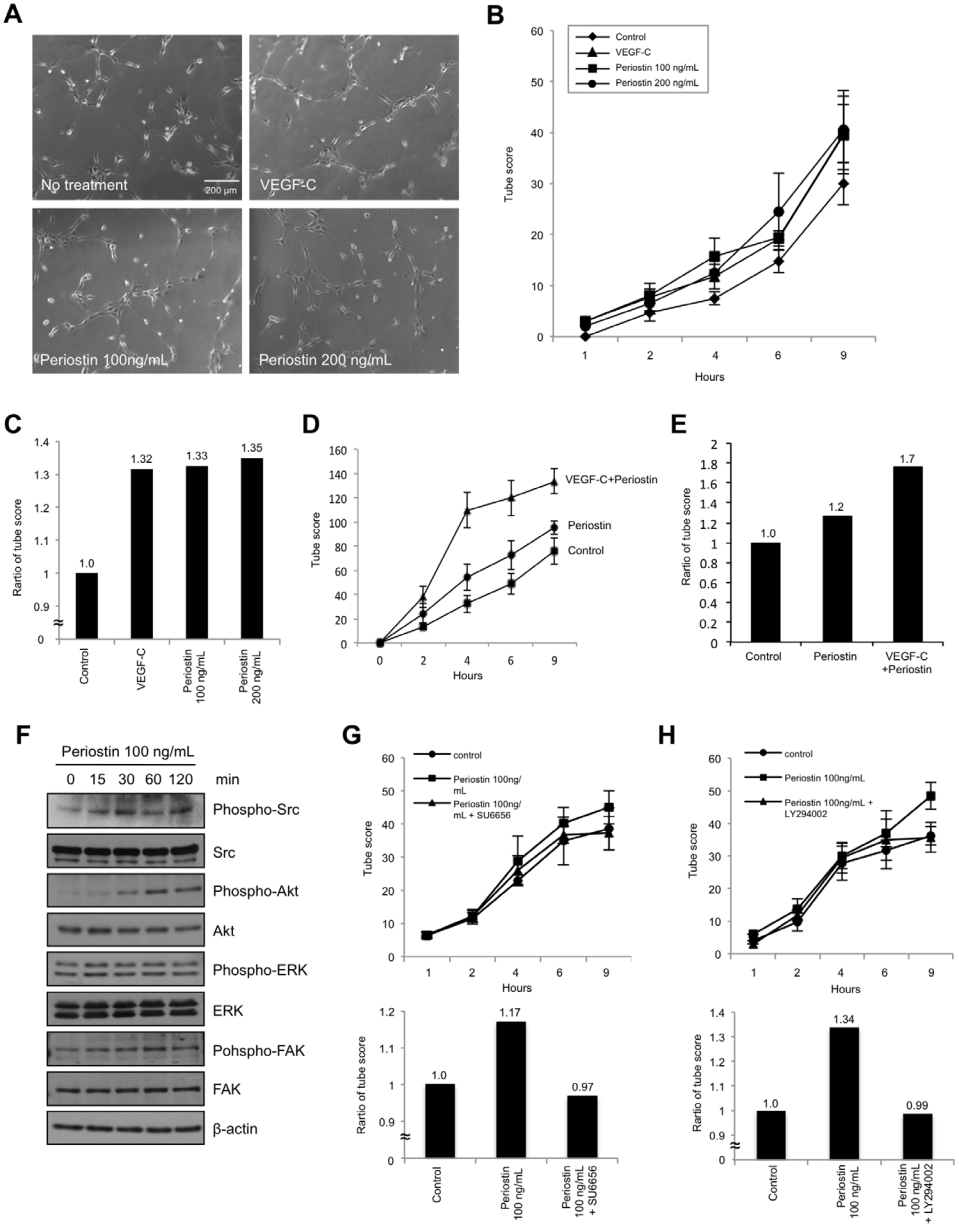
Direct involvement of periostin in tube formation of lymphatic endothelial cells. (A) TR-LE cells were seeded onto matrigel-coated wells in the presence of periostin (100 or 200 ng/mL) or VEGF-C (100 ng/mL). After incubation for 1–9 h, the lengths of the tube-like structures formed were evaluated. The figure shows cells after incubation for 9 h. (B) The graph shows the tube scores after incubation for 1–9 h. The bars show the average values and SDs from 3 independent experiments. (C) The graph shows the tube score ratios after treatment with VEGF-C or periostin for 9 h. The tube score of control was defined as 1.0. (D) TR-LE cells were seeded onto matrigel-coated wells in the presence of periostin (100 ng/mL) and VEGF-C (100 ng/mL). After incubation for 1–9 h, the lengths of the tube-like structures formed were evaluated. The graph shows the tube score after incubation for 1–9 h. The bars show the average values and SDs from 3 independent experiments. (E) The graph shows the tube score ratios after treatment with VEGF-C and periostin for 9 h. The tube score of control was defined as 1.0. (F) Periostin promotes Src and Akt phosphorylation. Levels of total and phosphorylated forms of Src, Akt, ERK, and FAK after treatment of TR-LE cells with periostin (100 ng/mL) shown by western blotting. β-actin expression was used as a loading control. (G) The lengths of the tube-like structures formed by TR-LE cells incubated for 1–9 h with recombinant periostin (100 ng/mL) with or without SU6656 (400 nM) were evaluated. The upper graph shows the tube scores after incubation for 1–9 h. The bars show the average values and SDs from 3 independent experiments. The lower graph shows the tube score ratio. The tube score of control was defined as 1.0. (H) The lengths of the tube-like structures formed by TR-LE cells incubated for 1–9 h with recombinant periostin (100 ng/mL) with or without LY294002 (10 µM) were evaluated. The upper graph shows the tube scores after incubation for 1–9 h. The bars show the average values and SDs from 3 independent experiments. The lower graph shows the tube score ratio. The tube score of control was defined as 1.0.

As shown above, we found that periostin expression correlated well with VEGF-C expression. Periostin has previously been shown to promote angiogenesis, as demonstrated by several findings: (i) periostin enhances VEGF receptor Flk-1/KDR expression in endothelial cells through an integrin αvβ3-FAK-mediated signaling pathway [13], (ii) recombinant periostin enhances capillary formation [12], and (iii) increased expression of periostin is correlated with the number of blood vessels and with metastasis in HNSCC [14]. On the other hand, clinico-pathological studies showed that periostin was well correlated with lymph node metastasis in various cancers [15]. These findings made us hypothesize that periostin might directly affect to lymphatic endothelial cells in similar to vascular endothelial cells. Therefore, we investigated whether periostin might directly promote lymphangiogenesis. We examined the effect of periostin on tube formation by TR-LE cells. In previous reports, 100 ng/mL of recombinant periostin protein was used for *in vitro* experiments [12], [13]. Shao et al. examined Flk1 expression after treatment with recombinant periostin (0, 50, 100 and 250 ng/mL). As 100 ng/mL of periostin remarkably upregulated Flk1 expression, they used 100 ng/mL of periostin in their studies. In our ELISA analysis, we detected 11.4 ng/mL of periostin in conditioned media from MSCC-Inv1 cells. In addition, we detected from 0 to 15.1 ng/mL of periostin in serum from HNSCC patient (Table S2). Although 100 ng/mL of periostin seems to be high concentration, we thought that concentration of periostin may be higher in local tumor area than in serum. In addition, we found that periostin promoted tube formation in a concentration dependent manner (0, 50, 100 and 200 ng/mL) (Figure S4), and the effect of 100 ng/ml of periostin on tube formation is remarkable (Figure 3A-C and Figure S4). Although 100 ng/ml of periostin seems to be higher level in comparison with physiological level, we used 100 ng/ml of periostin in the following studies. 100 ng/mL of VEGF-C was used as a positive control for lymphangiogenesis. In various studies, 100 ng/mL of VEGF-C was used for *in vitro* experiments [18]–[20]. Interestingly, treatment with recombinant periostin promoted tube formation relative to no treatment (Figure 3A and 3B). Surprisingly, the effect of periostin on tube formation was similar to that of VEGF-C (Figure 3C). Moreover, co-treatment with periostin and VEGF-C markedly promoted tube formation (Figure 3D and 3E).

We investigated how periostin promotes lymphangiogenesis by using western blotting with phosphorylation-specific antibodies to determine the activity levels of cell signaling molecules, such as Src, Akt, ERK and FAK, in periostin-treated TR-LE cells. Src and Akt but not ERK and FAK were activated by recombinant periostin treatment (Figure 3F). To confirm the role of Src and Akt in periostin promoted lymphangiogenesis, we used SU6656 to inhibit Src activity and LY294002 to inhibit Akt activity. These kinase inhibitors inhibited periostin-induced tube formation by TR-LE cells (Figure 3G and 3H), suggesting that periostin-induced lymphangiogenesis may be mediated by Src and Akt activity.

**Figure 4:**
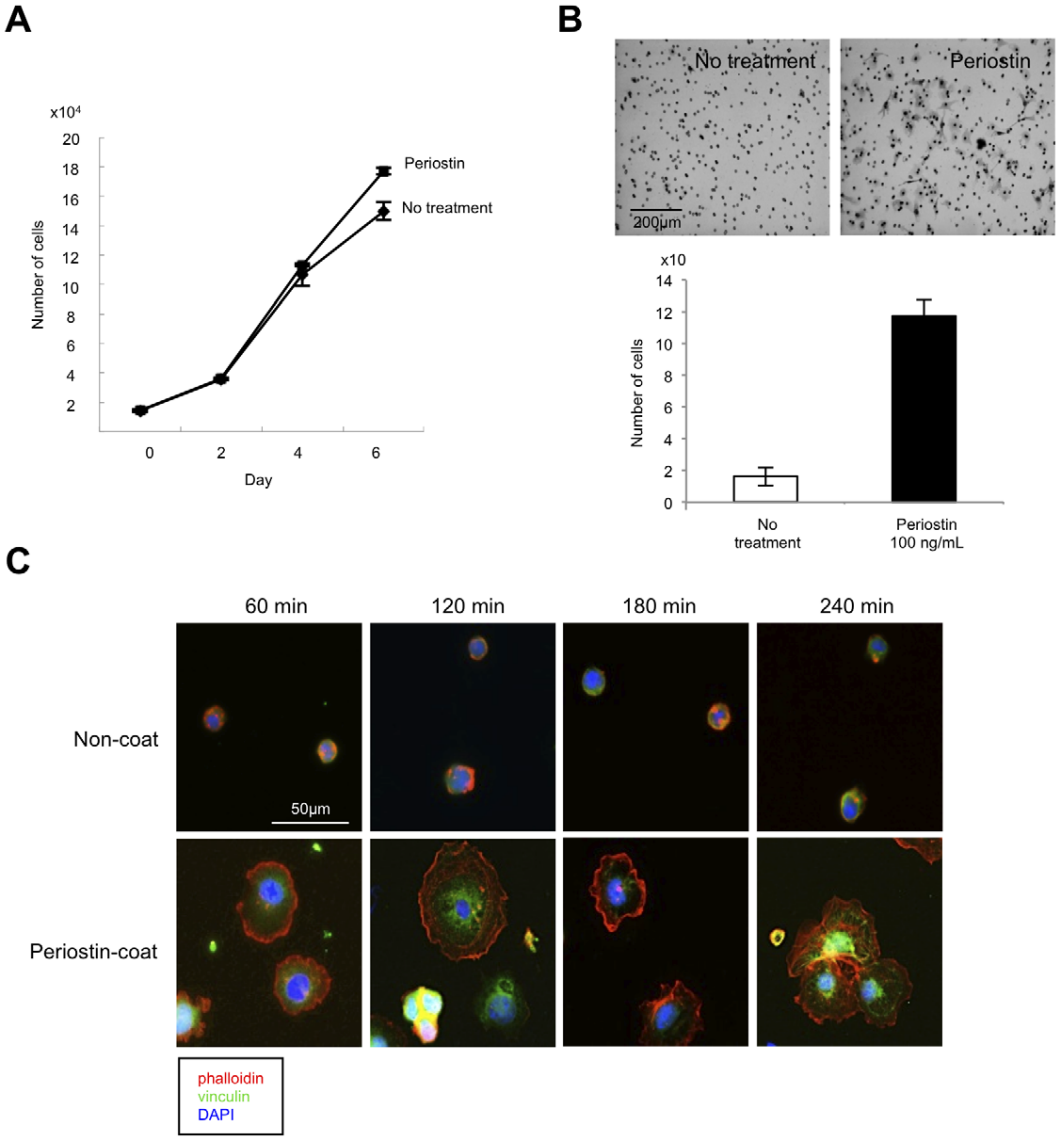
Periostin promotes proliferation and migration of lymphatic endothelial cells. (A) Proliferation of TR-LE cells after recombinant periostin treatment. Cells were seeded onto fibronectin-coated 24-well plates at 1.5×10^4^/well. After pre-incubation at 33°C for 24 h, the temperature was changed and recombinant periostin (100 ng/mL) was added to the medium. The cells were trypsinized and counted 0, 2, 4, or 6 days after the addition of recombinant periostin. The bars show the average values and SDs from 3 independent experiments. (B) The effect of periostin on cell migration of TR-LE cells. Cells were seeded onto filters pre-coated with 10 µg of fibronectin. The lower compartment contained 0.5 mL of serum-free medium with or without 100 ng/mL recombinant periostin. After incubation for 4 h, cells that had migrated to the lower surfaces of the filters were visualized by hematoxylin staining and counted. The assay was repeated 3 times. The figure shows cells that had migrated to the lower surface of the filter (upper panel). The graph shows the number of cells on the lower surfaces of the filters with or without periostin (100 ng/mL) (lower panel). (C) TR-LE cells were seeded on cover slips coated with PBS or recombinant periostin (200 ng/mL) and allowed to attach for 60, 120, 180, or 240 min. The cells were stained with Alexa Fluor 488-phalloidin antibody and anti-vinculin-FITC antibody. DNA was visualized by 4′,6-diamidino-2-phenylindole (DAPI) staining.

Next, we examined the effect of periostin on proliferation and migration of TR-LE cells. Recombinant periostin only slightly promoted cell growth (Figure 4A) but greatly promoted migration (Figure 4B). Focal adherens, which are clusters of integrins with accumulation of multiple cytoskeletal and signaling proteins around the integrin cytoplasmic domains, have significant effects on cell migration and signaling. Therefore, we examined the effect of periostin on the formation of focal adhesions. We seeded trypsinized TR-LE cells on cover slips coated with PBS or recombinant periostin and stained for focal adhesions with anti-vinculin antibody and for F-actin with phalloidin. TR-LE cells efficiently attached and rapidly recovered their morphology on periostin-coated cover slips compared with PBS-coated cover slips (Figure 4C). Notably, TR-LE cells formed numerous vinculin-containing focal adhesions on the periostin-coated but not the PBS-coated cover slips (Figure 4C). Enhancement of tube formation by periostin may be affected by promotion of migration and increased focal adhesions.

### Periostin expression correlates well with the number of lymphatic endothelial cells in clinical cancer specimens

**Figure 5:**
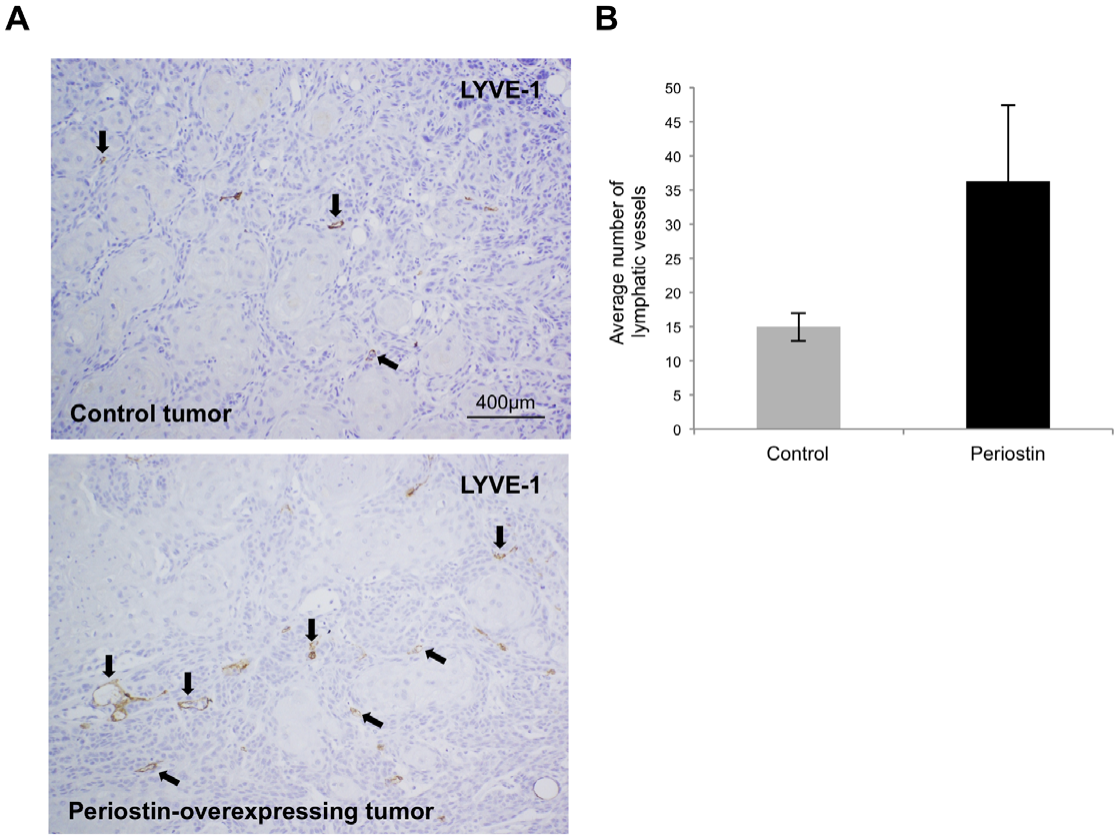
Periostin promotes lymphangiogenesis in vivo. (A) Periostin-overexpressing (periostin) and empty vector-transfected (control) HSC2 cells (1×10^7^ cells) were individually injected subcutaneously at 2 sites in each of 5 nude mice. After 1 month, the tumors were resected and stained with an anti-mouse LYVE-1 antibody recognising lymphatic vessels. Representative cases of immunohistochemical staining for LYVE-1 in control and periostin-overexpressing tumors are shown. Arrow shows LYVE-1 positive lymphatic vessels. (B) The LYVE-1 positive lymphatic vessels in control and periostin-overexpressing tumors were counted. The graph shows the average numbers of lymphatic vessels in control and periostin-overexpressing tumors. The bars show the average values and SDs.

To know the role of periostin in tumor lymphangiogenesis *in vivo*, we confirmed the correlation between periostin expression and the number of lymphatic vessels in a tumor xenograft model in nude mice. We previously injected control or periostin-overexpressing HNSCC cells subcutaneously into nude mice and found that transplanted periostin-overexpressing cells produced comparatively larger tumor volume after 28 days than did empty vector-transfected cells [9] (Figure S5). We used these tumor tissues (10 control and 10 periostin-overexpressing tumors) to count the numbers of lymphatic vessels expressing LYVE-1, which was specifically observed in lymph vessels in both control and periostin-overexpressing tumors (Figure 5A and Figure S4). Interestingly, the number of lymph vessels was significantly higher in periostin-overexpressing tumors (36.3±11.1) than in control tumors (14.95±2.0) (Figure 5B).

**Figure 6:**
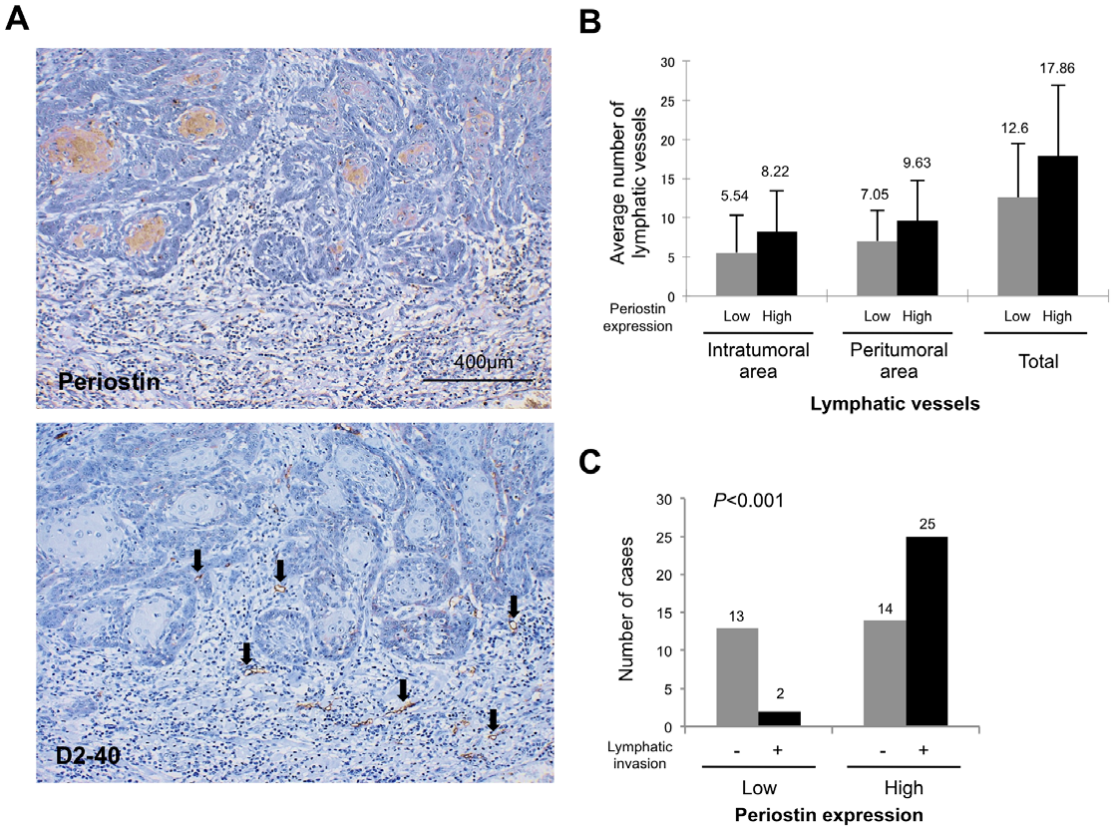
Correlation between periostin expression and lymphatic status in clinical HNSCC cases. (A) Periostin and D2-40 expression levels were examined by immunohistochemical staining of HNSCC case specimens. A representative case of periostin and D2-40 expression in HNSCC is shown. Arrow shows D2-40 positive lymphatic vessels. (B) The graph shows the comparison between the average numbers of lymph vessels in intratumoral, peritumoral, and total areas in HNSCC cases with high or low expression of periostin. (C) The graph shows the status of lymphatic invasion in HNSCC cases with high or low expression of periostin.

Finally, we examined the correlation between periostin expression and lymphatic status in HNSCC cases by immunohistochemistry using the D2-40 antibody. The D2-40 antibody specifically recognized lymphatic endothelial cells but not blood vessels (data not shown). Lymphatic vessels were unevenly distributed throughout the tumors. We counted the number of lymphatic vessels in the intratumoral (within the tumor) and peritumoral (within 1 mm of the invasive front) areas. HNSCC cases with periostin expression tended to have higher numbers of lymphatic vessels in both the intratumoral and peritumoral areas (Figure 6A and 6B), but this correlation was not statistically significant. As expected, D2-40 immunostaining highlighted the presence of lymphatic invasion. Of 54 HNSCC cases, 27 (50%) exhibited lymphatic vessel invasion by tumor cells. Strikingly, periostin expression was observed in 25 of 27 HNSCC cases with lymphatic invasion (Figure 6C). Periostin expression was significantly correlated with lymphatic invasion (*P*<0.001).

## Discussion

The overall 5-year survival rate for HNSCC, one of the most common cancers, is low, largely due to the propensity of some tumors to disseminate via the lymphatics. Indeed, lymph node involvement is one of the strongest poor prognostic indicators. Recent studies using animal models suggest that solid tumors can induce lymphangiogenesis that may in turn promote tumor spread [21]. Moreover, lymphangiogenesis can occur adjacent to or within cancers and correlates with lymph node metastasis [22], [23]. We have previously shown that the number of lymph vessels in both intratumoral and peritumoral areas was well associated with an increased tendency for nodal metastasis in HNSCC [24]. While lymphangiogenesis in primary tumors is known to predict nodal metastasis, its mechanism remains unclear.

We previously identified periostin as an invasion-promoting factor by comparing the gene expression profiles of parent and highly invasive clone HNSCC cells [9]. Periostin, originally called osteoblast-specific factor-2 (Osf2), was first identified in bone, where it was implicated in regulating osteoblast adhesion and differentiation [10], [25]. Cumulative evidence shows that high expression of periostin is frequently observed in various cancers and correlates well with malignant behavior [15]. Recently, periostin has been revealed to stimulate metastatic growth by inducing angiogenesis [13], [14]. Periostin secreted by tumor cells acts in a paracrine manner to augment the survival of endothelial cells and induces neovascularization, consistent with the notion that enhanced survival of intratumoral endothelial cells is critical for successful tumor angiogenesis [26]–[28]. Our previous microarray analysis of parent and highly invasive clone HNSCC cells found that VEGF-C was upregulated in the highly invasive cells [9] (Figure 1A and 1B). Indeed, periostin overexpression induced the upregulation of VEGF-C expression in HNSCC cells, and VEGF-C protein was secreted from periostin-overexpressing HNSCC cells (Figure 1B). VEGF-C, the original Flt-4/VEGFR-3 ligand [29], [30], is a member of the VEGF family of polypeptide growth factors, which comprises VEGF-A, -B, -C, -D and the parapoxvirus Orf virus VEGFs [31], [32]. Based on its expression profile and its binding to Flt-4, VEGF-C has been implicated in the development of the lymphatic system [33], [34]. Moreover, transgenic overexpression of VEGF-C under the keratin 14 promoter induces lymphatic vessel enlargement in the skin [35], and recombinant VEGF-C induces lymphangiogenesis in the chick chorioallantoic membrane [36]. Activation of the VEGF-C/Flt-4 axis in lymphatic endothelial cells can facilitate metastasis by increasing the formation of lymphatic vessels within and around tumors [37]. The VEGF-C/Flt-4 axis is therefore expressed not only by lymphatic endothelial cells but also by a variety of human tumor cells. As we expected, conditioned media from periostin-overexpressing HNSCC cells promoted tube formation of lymphatic endothelial cells. Moreover, the VEGFR-3 kinase inhibitor inhibited the tube formation induced by conditioned medium from periostin-overexpressing cells, suggesting that periostin-promoted lymphangiogenesis may be caused in part by increased secretion of VEGF-C from cancer cells (Figure S2B). By using clinical samples, we demonstrated that periostin expression correlated well with VEGF-C expression in HNSCC cases by immunohistochemistry and that serum periostin level correlated well with that of VEGF-C in HNSCC patients (Figure 2E). In this study, we could not detect periostin in serum from 5 normal healthy volunteers. Previous reports showed that wild range of serum periostin (0.2–194 ng/mL) was detected in normal healthy volunteers [35]–[38]. Thus, serum level of periostin in normal healthy volunteers seems to be controversial, and it is still unclear whether periostin is produced by normal cells or not. In the future, we will examine the serum level of periostin in the large number of normal healthy volunteers. However, all reports including the present study showed that increased level of periostin was observed in cancer patients [38]–[41]. Importantly, serum level of periostin and VEGF-C was well correlated with tumor progression including lymph node metastasis. Although we have to examine the relationship between protein-expression level of primary tumor and serum level in a large number of HNSCC cases, detection of serum level of periostin and VEGF-C may be a useful for prediction of malignant behaviors of HNSCC patients.

Surprisingly, recombinant periostin directly promoted tube formation by lymphatic endothelial cells. Moreover, periostin activated Src and Akt in TR-LE cells (Figure 3F) and inhibition of Src or Akt inhibitor suppressed tube formation driven by periostin (Figure 3G and 3H). Previous studies show that c-Src regulates the formation of the complex between VEGFR-2 and integrin αvβ3 by phosphorylating the cytoplasmic tyrosine residues of integrin β3 inducing structural changes on the complex that promote capillary formation and chemotaxis [42], [43], and that c-Src phosphorylates the C-terminal domain of VEGFR-3 at several residues including those critical for the activation of downstream signaling [44]. We previously found that interference with the function of integrins by specific anti-αvβ3 and anti-αvβ5 integrin antibodies had an effect on the ability of periostin to mediate cell adhesion in HNSCC cells [9]. Moreover, previous report that periostin activated the Akt/PKB pathway via the αvβ3 integrin to promote cellular survival in colon cancer [12]. Therefore, these finding suggest that periostin-integrin interaction may trigger the intracellular signaling and activation of certain genes that are involved in tube formation and migration of lymphatic endothelial cells through Src and Akt activation. Indeed, several recent reports show that Src and Akt are involved in lymphangiogenesis: (i) loss of Akt1 reduces lymphatic capillary size and causes defects in the maturation of collecting lymphatic vessels and in valve development [45], (ii) VEGFR-3 mediates activation of Akt as well as the Erk1/2 pathways in primary lymphatic endothelial cells [46] and (iii) inhibition of Src kinase is strongly anti-lymphangiogenic *in vitro* and *in vivo* [47]. In addition to promoting tube formation, periostin promoted migration and formation of focal adherens (Figure 4B and 4C). These phenotypes may play an important role in periostin-mediated lymphangiogenesis. To our knowledge, this is the first report containing *in vitro* data on the roles of periostin in tumor lymphangiogenesis. However, detailed molecular mechanism of periostin-driven lmphangiogenesis is still unclear. In endothelial cells, periostin promotes angiogenesis via the upregulation of Flk-1/KDR expression through the integrin αvβ3-FAK-mediated signaling pathway [13]. Therefore, we hypothesized that periostin might upregulate expression of the VEGF-C receptor Flt-4 in lymphatic endothelial cells; however, this was not the case (Figure S2A and S2B). Although ectopic overexpression of periostin induced upregulation of VEGF-C in HNSCC cells, treatment with recombinant periostin did not induce upregulation of VEGF-C in lymphatic endothelial cells (Figure S6A and S6B). This discrepancy may be accounted by the cell type. A previous report shows that proinflammatory cytokines such as IL-1α, IL-1β, and TNF-α upregulate VEGF-C mRNA expression [48]. However, the detailed mechanism of VEGF-C induction remains unclear, making it interesting to elucidate the mechanism by which periostin upregulates VEGF-C.

Increased number of lymph vessels was observed in xenograft tumors of periostin-overexpressing HNSCC cells (Figure 5A and 5B), and periostin expression positively correlated with the number of lymph vessels and with lymphatic invasion in HNSCC cases (Figure 6A–C). In non-small-cell lung cancer, periostin expression also correlates well with lymphatic microvessel density [49]. Moreover, several immunohistochemical studies have found possible correlations between periostin expression and lymph node metastasis in cancer cases including HNSCC, gastric cancer cholangiocarcinoma, thyroid cancer, pancreatic cancer and melanoma [9], [14], [50]–[54]. It is interesting to examine the correlation between periostin and lymhangiogenesis in these types of cancer.

**Figure 7:**
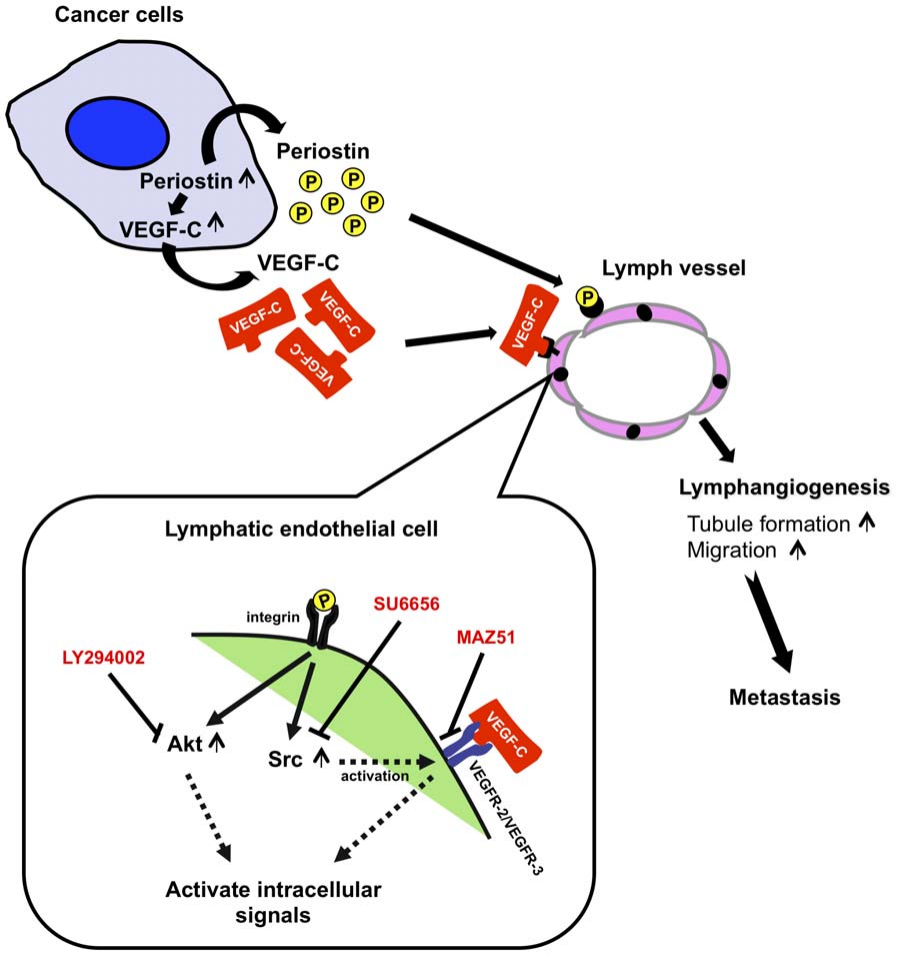
A model of periostin-promoted lymphangiogenesis. Periostin expression is upregulated in cancer cells. Periostin in turn upregulates VEGF-C expression in cancer cells. Periostin secreted from the cancer cells promotes migration and tube formation of lymphatic endothelial cells through the activation of Src and Akt. Src may activate VEGFR-2 and VEGFR-3 [36]–[38]. Moreover, Secreted VEGF-C from cancer cells also promotes migration and tube formation of lymphatic endothelial cells. Thus, in addition to activation of VEGF-C signaling pathway, periostin-integrin interaction may trigger the intracellular signaling and activation of certain genes that are involved in tube formation and migration of lymphatic endothelial cells through Src and Akt activation.

In conclusion, we have demonstrated that periostin has a novel function as a direct and/or indirect promoter of tumor lymphangiogenesis. Periostin-promoted lymphangiogenesis was mediated by increased secretion of VEGF-C from cancer cells and by migration and tube formation via activation of Src and Akt in lymphatic endothelial cells (Figure 7). Importantly, the serum periostin level in HNSCC patients correlated well with that of VEGF-C and with malignant behaviors including increased tumor stage and lymph node metastasis. Therefore, periostin could be a marker for prediction of malignant behaviors in HNSCC. Our present and previous findings reveal that periostin may play important roles in metastasis through promotion of invasion, angiogenesis, and lymphangiogenesis. We conclude that periostin may be a potential target for future therapeutic intervention to obstruct tumoral lymphatic invasion, angiogenesis, and lymphangiogenesis in HNSCC patients.

## Materials and Methods

### Reagents

Human recombinant VEGF-C protein was obtained from R&D Systems (Minneapolis, MN). Human recombinant periostin protein was obtained from PROSPEC (East Brunswick, NJ). The inhibitors of VEGF receptor 3 kinase (MAZ51), Src (SU6656), and PI3 Kinase (LY294002) were obtained from Calbiochem (San Diego, CA).

### Cell culture

MSCC-1 and MSCC-Inv1 cells were previously established in our laboratory [7], [8] and were maintained in Keratinocyte-SFM (Invitrogen, San Diego, CA) under 5% CO_2_ in room air at 37°C. MSCC-1 cells were established from lymph node metastatic tumor tissue of a patient with gingival squamous cell carcinoma [7]. MSCC-Inv1 cells were isolated from MSCC-1 cells by using an *in vitro* invasion assay device [8]. HNSCC cell lines HSC4 and Ca9-22 were provided by the Japanese Collection of Research Bioresources Cell Bank and maintained in RPMI-1640 (Nissui Pharmaceutical Co., Tokyo, Japan) supplemented with 10% heat-inactivated foetal bovine serum (FBS) (Invitrogen) and 100 U/mL penicillin-streptomycin (Invitrogen) under 5% CO_2_ in room air at 37°C. On receiving the cell lines, they were immediately cultured and expanded to prepare frozen ampule stocks. Cells were passaged for no more than 2 to 3 months before establishing new culture from early-passage frozen ampules. Periostin-overexpressing HSC4 and Ca9-22 cells were previously obtained by transfection with a retroviral plasmid encoding hexa-histidine-tagged periostin [14]. Immortalized rat lymphatic endothelial cell line, TR-LE cell was established from thoracic duct of tsA58 transgenic rats [16]. They were maintained on culture dishes pre-coated with 10 µg/mL fibronectin (Iwaki Glass, Tokyo, Japan) in HuMedia-EG2 (Kurabo, Osaka, Japan) supplemented with 10% FBS at a permissive temperature (33°C). For the growth assay, the cells were plated onto 24-well plates (Falcon Becton Dickinson, Franklin Lakes, NJ), trypsinized, and counted using a Cell Counter (Coulter Z1, Coulter Co., Hialcah, FL). All of the cell lines used the course of these studies were maintained in continuous culture for 3 months or less.

### Reverse-transcription polymerase chain reaction (RT-PCR)

Total RNA was isolated from tumor tissues and cell lines using the RNeasy Mini Kit (Qiagen, Hilden, Germany). RNA concentration was quantified and its purity determined by standard spectrophotometric methods. cDNA was synthesized from 1 µg total RNA using ReverTra Dash (Toyobo Biochemicals, Tokyo, Japan) according to the manufacturer's instructions. The primer pair sequences used were: human periostin, 5′-gatggagtgcctgtggaaat-3′ (forward) and 5′-aacttcctcacgggtgtgtc-3′ (reverse) (product size: 239 bp); human VEGF-C, 5′-ggaaagaagttccaccacca-3′ (forward) and 5′-tttgttagcatggacccaca-3′ (reverse) (product size: 249 bp); human GAPDH, 5′-accacagtccatgccatcac-3′ (forward) and 5′-tccaccaccctgttgctgta-3′ (reverse) (product size: 452 bp). Total cDNA was amplified using Go Taq® Green Master Mix (Promega, Madison, WI) in a PC701 thermal cycler (Astec, Fukuoka, Japan) for 25–30 cycles of denaturation at 94°C for 20 s, annealing at 60°C for 30 s, and extension at 72°C for 1 min (for all primers). The amplicons were resolved on 1.5% agarose/TAE gels (Nacalai tesque, Inc., Kyoto, Japan) at 100 mV and visualized by ethidium bromide staining.

### Western blot analysis

Western blotting was performed as previously described [9]. Sample protein concentrations were measured by Bradford protein assay (Bio-Rad, Richmond, CA), and 20 µg total protein/lane subjected to electrophoresis on 10% polyacrylamide gels followed by electroblotting onto nitrocellulose filters. The membranes were blocked with 3% milk in TBS-T and incubated overnight at 4°C with the following antibodies: anti-β-actin monoclonal antibody (Sigma, St. Louis, MO), anti-His-tag polyclonal antibody (Cell Signaling Technology, Beverly, MA), anti-phospho-FAK (Tyr576/Tyr577) monoclonal antibody (Cell Signaling Technology), anti-phospho-Akt (Ser473) monoclonal antibody (Cell Signaling Technology), anti-phospho-Src (Tyr416) polyclonal antibody (Cell Signaling Technology), anti-phospho-ERK monoclonal antibody (Santa Cruz Biotechnology, Inc.), anti-FAK polyclonal antibody (Cell Signaling Technology), anti-Akt polyclonal antibody (Cell Signaling Technology), anti-Src polyclonal antibody (Cell Signaling Technology), and anti-ERK monoclonal antibody (Cell Signaling Technology). The membranes were then washed with TBS-T and incubated with specific secondary antibodies and the proteins visualized using the ECL western blotting detection system (Amersham, Piscataway, NJ).

### Patient samples

Archived paraffin-embedded tissue specimens from 54 previously untreated HNSCC patients were obtained from the Department of Oral Pathology, Faculty of Dental Sciences, University of Peradeniya, Sri Lanka after approval by the Ethical Committees of all involved institutions. Clinical information including age, sex, location and lymphatic invasion was gathered from surgical records of the patients (Table S1). All received surgery as their first-line treatment, with some receiving postoperative radiotherapy.

Peripheral blood was collected from 81 current HNSCC cancer patients. Forty-four samples were obtained from the Department of Oral and Maxillofacial Sciences, Gifu University Graduate School of Medicine and 37 from Cancer Hospital in Ho Chi Minh (Vietnam) after approval by the Ethical Committees of the respective institutions. Blood samples were centrifuged and plasma separated and frozen in aliquots at -80°C for later analysis. Clinical information including lymph node metastasis, tumor stage and TNM classification was gathered from surgical records of the patients (Table S2).

### Immunohistochemical staining

The tumor tissues were fixed in 10% formalin, embedded in paraffin, and cut into 4-µm-thick sections. The sections were stained with hematoxylin and eosin (H&E) for histological examination. Immunohistochemical detection of periostin, VEGF-C and D2-40 in HNSCC cases was performed on 4.5 µm sections mounted on silicon-coated glass slides, using the EnVision system (DAKO, Glostrup, Denmark). A polyclonal anti-Periostin antibody was generated by immunizing the rabbits with specific peptides (EGEPEFRLIKEGETC) for periostin and purified through an affinity column. We previously confirmed the specificity of this antibody by recognizing a recombinant Periostin protein, ectopic overexpressing His-tagged periostin in lysates and secreted Periostin protein in a conditioned media from periostin-overexpressing cells [15]. Anti-D2-40 monoclonal antibody (Signet, Dedham, MA; dilution 1∶40) and anti-VEGF-C polyclonal antibody (C-20, Santa Cruz Biotechnology, Santa Cruz, CA; dilution 1∶25) were also used. The specific secondary antibody was then added and visualized with diaminobenzidine (DAB). For the evaluation of staining intensity, we graded + (weak/focal immunopositivity), ++ (strong/focal immunopositivity and weak/diffuse immunopositivity) and +++ (strong/diffuse immunopositivity). Then, the expression of periostin and VEGF-C was graded as high (over 10% of tumor cells showing ++ or +++ intensity) or low (no staining or less than 10% of tumor cells showing + intensity).In HNSCC cases with the heterogenous staining, we observed at least ten fields including superficial, central and deep invasive areas of the tumor. Then we totally evaluated the number of stained cells and their staining intensity as described above. For periostin expression, as periodontal ligament expresses periostin [10], we used a specimen of gingival cancer cases with periodontal tissue as an internal positive control (Figure S1A). When the slides were visualized with DAB staining, we used this specimen as a positive control for assessment of intensity of staining. Negative controls were processed as above except for the primary antibody was used. In addition, as several slides of HNSCC cases include the normal oral mucosa, we used the staining of normal epithelial cells as a negative control of periostin (Figure S1B). For VEGF-C expression, specificity of antibody was checked by staining without primary antibody. Moreover, we confirmed the positivity by using this antibody in VEGF-C positive HNSCC cell line by immunohistochemistry (data not shown). One specimen of HNSCC case with VEGF-C positive staining was used for assessment of intensity of staining. As several slides of HNSCC cases include the normal oral mucosa, we used the staining of normal epithelial cells as a negative control of VEGF-C.

### Evaluation of intratumoral and peritumoral lymphangiogenesis

We used one section (4.5 µm) of each tumor. After staining with D2-40, We counted the number of lymphatic vessels in 6 high-power fields (×100) with the highest lymphatic vascular density (hot spots); within the tumor and within an area 1 mm from the tumor border (along the invasive front) in each tumor section. Each selected field was micro-photographed and the positively stained lymph vessels traced using Adobe Photoshop software. Total vessel counts averaged over the 6 high-power fields (×100) were obtained from each tumor. For evaluating the invasion of tumor cells into lymphatic vessels, the whole tumor area was scanned.

### ELISA

Periostin levels were determined using sandwich enzyme-linked immunosorbent assay (ELISA) kits provided by Immuno-Biological Laboratories Co., Ltd. (Gunma, Japan). The VEGF-C levels were determined using commercially available sandwich ELISA kits (human VEGF-C ELISA kit; Immuno-Biological Laboratories Co., Ltd.). Briefly, serum samples were incubated in a micro-well plate pre-coated with rabbit anti-human periostin IgG or rabbit anti-human VEGF-C IgG. Any target protein present in the samples thus bound to the wells, and the excess was removed by extensive washing. The amount of periostin or VEGF-C was measured using a peroxidase-conjugated secondary antibody detected by the addition of tetramethylbenzidine (TMB). The reactions were stopped with an acid solution and the optical density at 450 nm read in a micro-titer plate spectrophotometer. The serum target protein concentrations were determined from corresponding standard curves run separately for each plate.

### Tube formation assay

Sub-confluent TR-LE cells were harvested with trypsin-EDTA, centrifuged at 1200 rpm for 5 min, suspended (6×10^4^) in HuMedia-EG2 (Kurabo) without growth factors or FBS, and seeded in a 24-well plate pre-coated with 200 µL of 10 mg/mL matrigel (Falcon Becton Dickinson). After incubation at 37°C for 1–9 h, the cells were photographed with an epifluorescence Zeiss Axioplan 2 (Zeiss Inc., Thorwood, NY) microscope attached to a CCD camera. The lengths of the tube-like structures formed were measured by Chalkley Counting modified. Briefly, the four most vascular areas (hot spots) with the highest number of microvessel profiles were chosen subjectively from each well. A 25-point Chalkley eyepiece graticule was applied to each hot-spot area and oriented to permit the maximum number of points to hit on, or within the areas. The Chalkley count is the number of grid points that hit microvessels. The Chalkley count for an individual well was taken as the mean value of the four graticule counts. All Chalkley counts were performed by one observer and used the microscope with the lowest maginification.

### Migration assay

Cell migration activity was measured using 24-well cell culture inserts with 8-µm pores (3097, Falcon Becton Dickinson). The filters were coated with 10 µg of fibronectin (Iwaki Glass) for 12 h at 4°C. Each lower compartment contained 0.5 mL of serum-free medium with or without 100 ng/mL recombinant periostin. After trypsinisation, 5×10^4^ cells were resuspended in 100 µL of serum-free medium and placed in the upper compartment of the cell culture insert for 4 h. To examine the migration activity, the cells that had penetrated onto the lower side of the filter were fixed with formalin and stained with hematoxylin. We counted total number of the penetrated cells in each assay. The assay was repeated 3 times.

### Immunofluorescence analysis

TR-LE cells were seeded on cover slips coated with phosphate-buffered saline (PBS) or recombinant periostin (200 ng/mL) and allowed to attach for 60, 120, 180, or 240 min. The cover slips were washed with PBS and fixed with 4% paraformaldehyde at room temperature for 10 min, rinsed 3 times with PBS, and then permeabilized in 0.1% Triton X-100 in PBS for 15 min at room temperature. After 3 washes with PBS, the cover slips were incubated in PBS with Alexa Fluor 488-phalloidin (Molecular Probes, Eugene, OR) and anti-vinculin-FITC (Sigma). DNA was visualised by 4′,6-diamidino-2-phenylindole (DAPI) staining. The cell preparations were imaged using an epifluorescence Zeiss Axioplan 2 microscope (Zeiss, Inc.) attached to a CCD camera.

### Number of lymphatic vessels in Xenograft model

The anchorage-independent growth of periostin-overexpressing HNSCC cells in vivo was examined previously [9]. Periostin-overexpressing HSC2 cells (1×10^7^ in 500 µL of Hanks' balanced salt solution) were injected subcutaneously (s.c.) at multiple sites in athymic (nude) mice. The control mice were injected with the same number of vector-transfected HSC2 cells. The experimental protocol was approved by the Committee of Research Facilities for Laboratory Animal Science of Hiroshima University. The animals were monitored for tumor formation every week and sacrificed after 1 month. The tumor tissues (10 control and 10 pereiostin-overexpressing tumors) were fixed in 10% formalin, embedded in paraffin, and cut into 4-µm-thick sections. The sections were stained with anti-mouse LYVE-1 antibody (RELIA Tech GmbH, Wolfenbüttel, Germany) and the numbers of lymphatic vessels counted. For the evaluation of lymphangiogenesis, we counted and calculated the average number of lymphatic vessels of over 3 high-power fields (×100) in each tumor.

### Statistical analysis

Possible associations between parameters of the analyzed tumor samples were tested using the t-test. For the correlation between vessel infiltration and lymph node status and for the histopathological grading patterns, statistical significance was measured by the Chi-square test (χ^2^). For the correlation between periostin expression and lymph vessel density, statistical significance was measured by the Welch test. For the correlation between periostin expression and VEGF-C, statistical significance was measured by the Chi-square test (χ^2^). For all tests, a P-value <0.05 was considered statistically significant.

## Supporting Information

Figure S1 **Immunohistochemical staining of periostin.** **A:** Figure shows periostin expression in periodontal ligament. **B:** Figures show negative expression of periostin in normal oral mucosa associated to periostin positive HNSCC cases. Dotted line separates normal area (normal oral mucosa) without periostin expression and cancer area (moderately or poorly differentiated squamous cell carcinoma) with high expression of periostin.
(TIFF)

Figure S2 **VEGFR-3 kinase inhibitor suppresses tube formation by conditioned medium from periostin-overexpressing cells.** **A:** TR-LE cells were seeded onto matrigel-coated wells in the presence of conditioned medium from empty vector-transfected (Control CM), periostin-overexpressing (periostin CM) HSC4 cells or recombinant VEGF-C (100 ng/mL) with or without the VEGFR-3 kinase inhibitor, MAZ51 (1 µM). After incubation for 0–9 h, the lengths of the tube-like structures formed were evaluated. Figure shows the cells after incubation for 9 h. **B:** The graph shows the tube scores after incubation for 0–9 h. The bars show the average values and standard deviations (SDs) from 3 independent experiments. **C:** The graph shows the inhibitory ratio of tube formation by MAZ51 after 9 h (%) (right lower panel). The inhibitory ratio of tube formation was calculated as ‘100 - (tube score after treatment with MAZ51/tube score before treatment with MAZ51) ×100’.
(TIFF)

Figure S3 **Immunohistochemical staining of periostin in HNSCC cases.** **A:** A representative HNSCC cases of low expression of Periostin and VEGF-C. **B:** Figure shows heterogenous staining of periostin in HNSCC case with high expression of periostin. Dotted line shows a representative area of low expression of periostin.
(TIFF)

Figure S4 Direct involvement of periostin in tube formation of lymphatic endothelial cells. TR-LE cells were seeded onto matrigel-coated wells in the presence of periostin (0, 50, 100 or 200 ng/mL). The graph shows the tube score ratios after treatment with periostin for 9 h. The tube score of control was defined as 1.0.
(TIFF)

Figure S5 **Number of lymphatic vessels in xenograft periostin-overexpressing tumors.** Schema shows the method to count the number of lymphatic vessels in control and periostin-overexpressing tumors in xenograft model.
(TIFF)

Figure S6 **Periostin did not affect to Flt-4 expression. A:** Expression of Flt-4 and VEGF-C mRNA was examined by RT-PCR in TR-LE cells after 24 and 48 hr of Periostin treatment (100 ng/mL). GAPDH expression was used as a loading control. Two pairs of primer sequences were; rat Flt-4, 5′-taaccgacctcctggtgaac-3′ (forward) and 5′-tgcacacactgcacaggtaa-3′ (reverse) (product size, 204 bp); rat VEGF-C, 5′-agcagccacaaacaccttct-3′ (forward) and 5′-ttagctgcctgacactgtgg-3′ (reverse) (product size, 285 bp); rat GAPDH, 5′-accacagtccatgccatcac-3′ (forward) and 5′-tccaccaccctgttgctgta-3′ (reverse) (product size, 452 bp). **B:** Expression of Flt-4 and VEGF-C mRNA was examined by RT-PCR in TR-LE cells after 24 hr of Periostin treatment (100, 200 and 400 ng/mL), VEGF-C treatment (500 ng/mL) and Periostin (100 ng/mL) and VEGF-C (500 ng/mL) treatment.
(TIFF)

Table S1 **Clinical data of HNSCC patients and immunohistochemical results.**
(TIFF)

Table S2 **Clinical data of HNSCC patients and ELISA result.**
(TIFF)
