# Supplementary figures and images for: Periostin Directly and Indirectly Promotes Tumor Lymphangiogenesis of Head and Neck Cancer
Source: PLoS One. 2012 Aug 30;7(8):e44488. doi: 10.1371/journal.pone.0044488 (PMC3431354; doi:10.1371/journal.pone.0044488)

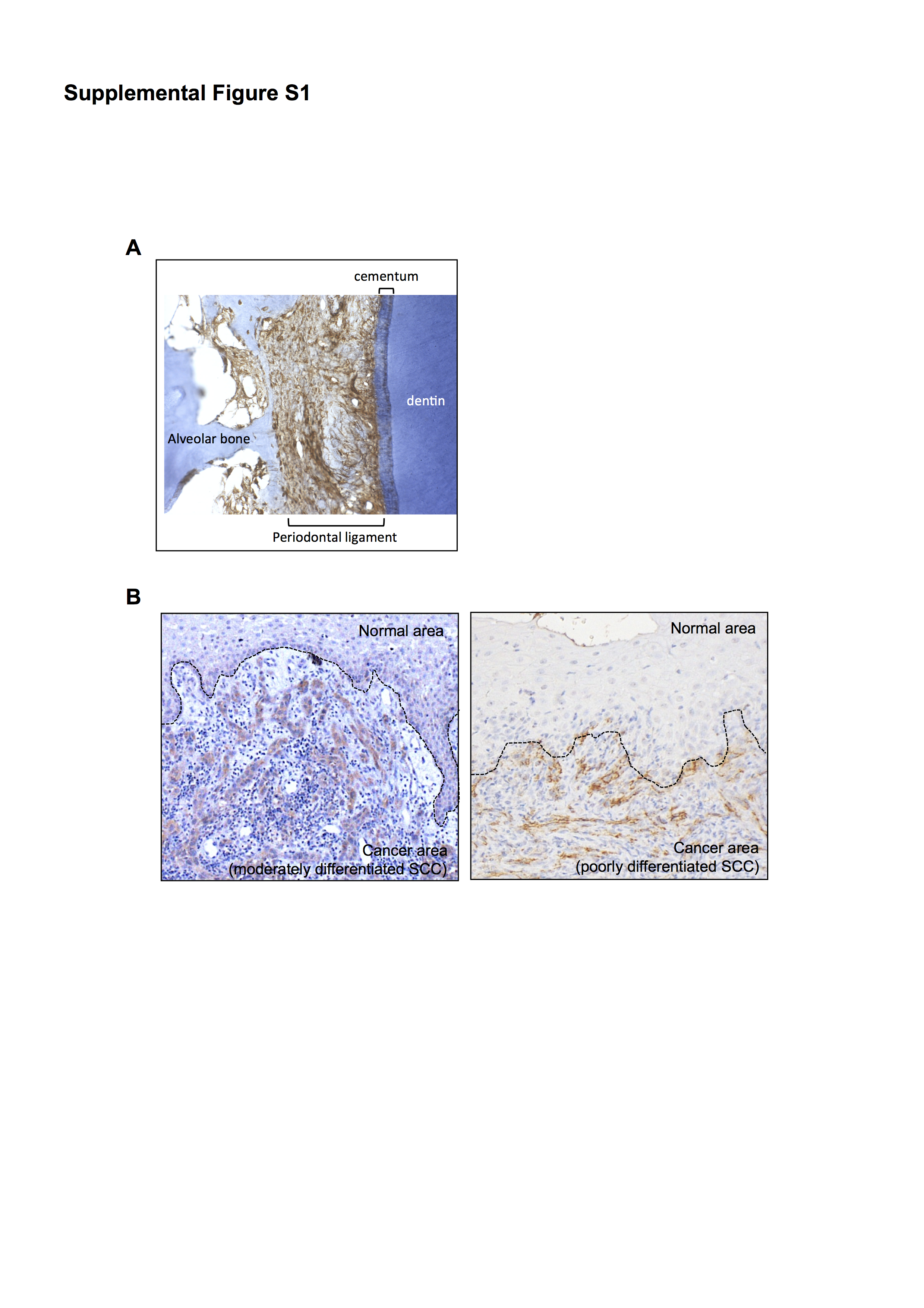

Supplement: Figure S1 — Immunohistochemical staining of periostin. A: Figure shows periostin expression in periodontal ligament. B: Figures show negative expression of periostin in normal oral mucosa associated to periostin positive HNSCC cases. Dotted line separates normal area (normal oral mucosa) without periostin expression and cancer area (moderately or poorly differentiated squamous cell carcinoma) with high expression of periostin. (TIFF) [file pone.0044488.s001.tiff]

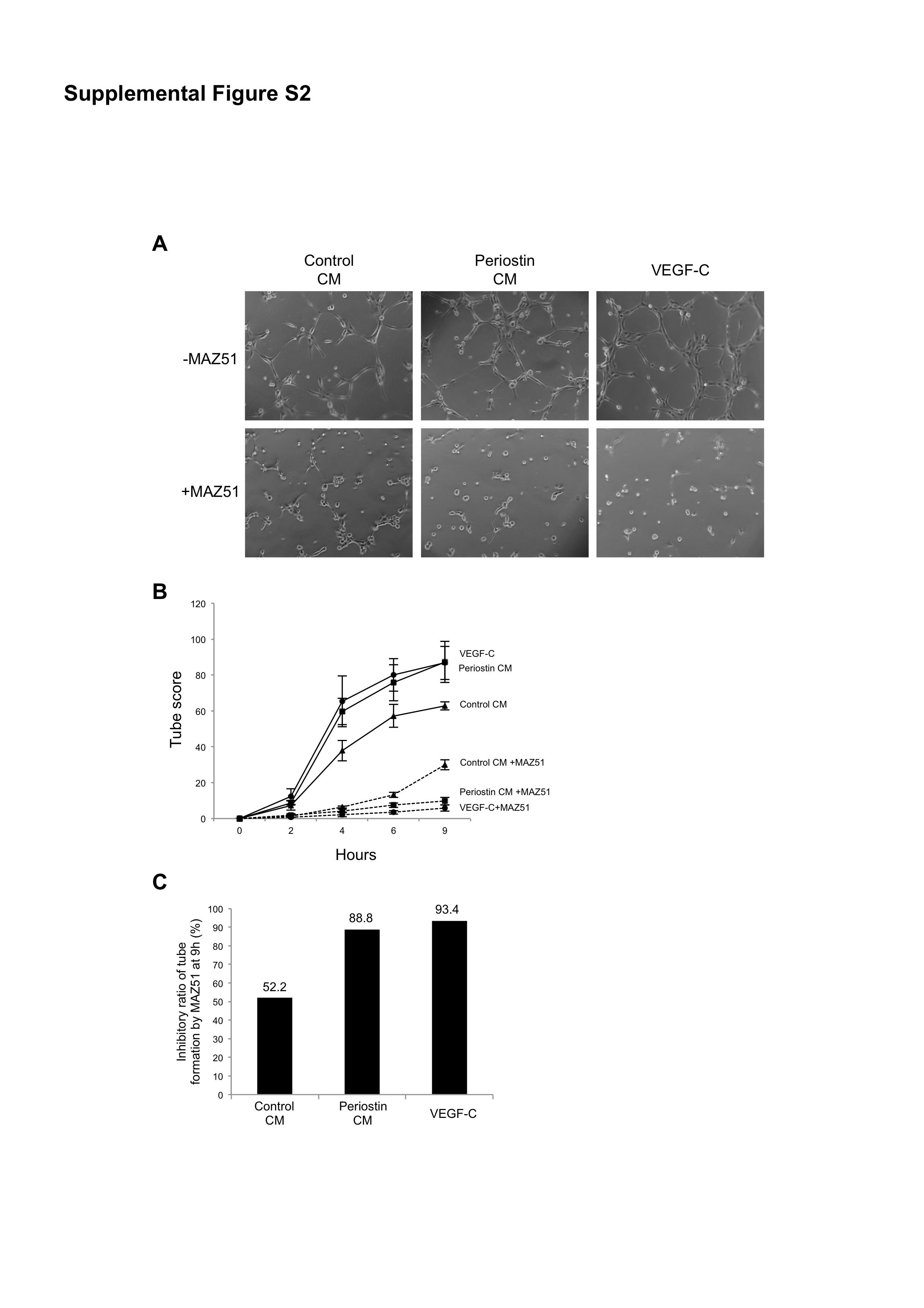

Supplement: Figure S2 — VEGFR-3 kinase inhibitor suppresses tube formation by conditioned medium from periostin-overexpressing cells. A: TR-LE cells were seeded onto matrigel-coated wells in the presence of conditioned medium from empty vector-transfected (Control CM), periostin-overexpressing (periostin CM) HSC4 cells or recombinant VEGF-C (100 ng/mL) with or without the VEGFR-3 kinase inhibitor, MAZ51 (1 µM). After incubation for 0–9 h, the lengths of the tube-like structures formed were evaluated. Figure shows the cells after incubation for 9 h. B: The graph shows the tube scores after incubation for 0–9 h. The bars show the average values and standard deviations (SDs) from 3 independent experiments. C: The graph shows the inhibitory ratio of tube formation by MAZ51 after 9 h (%) (right lower panel). The inhibitory ratio of tube formation was calculated as ‘100 - (tube score after treatment with MAZ51/tube score before treatment with MAZ51) ×100’. (TIFF) [file pone.0044488.s002.tiff]

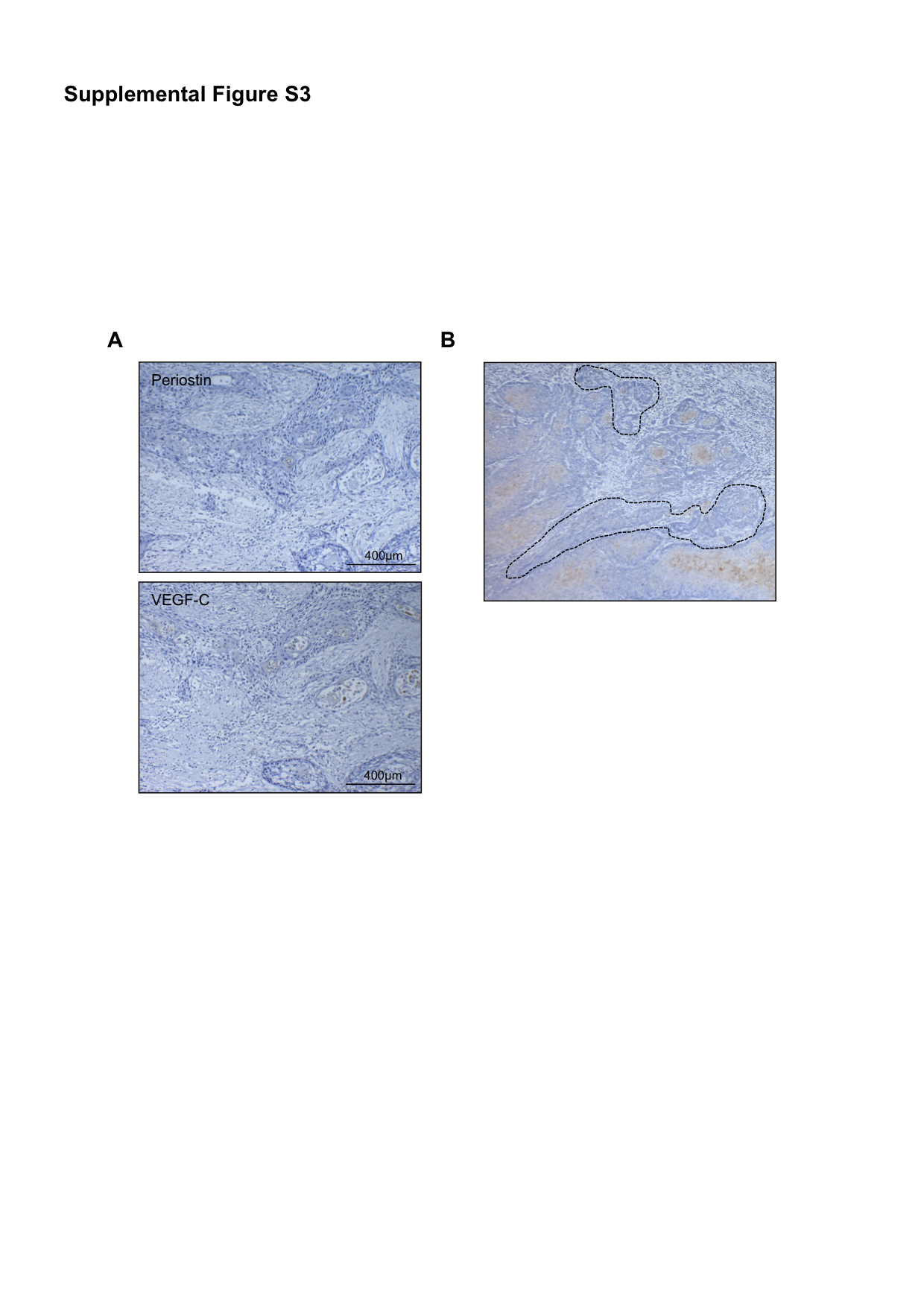

Supplement: Figure S3 — Immunohistochemical staining of periostin in HNSCC cases. A: A representative HNSCC cases of low expression of Periostin and VEGF-C. B: Figure shows heterogenous staining of periostin in HNSCC case with high expression of periostin. Dotted line shows a representative area of low expression of periostin. (TIFF) [file pone.0044488.s003.tiff]

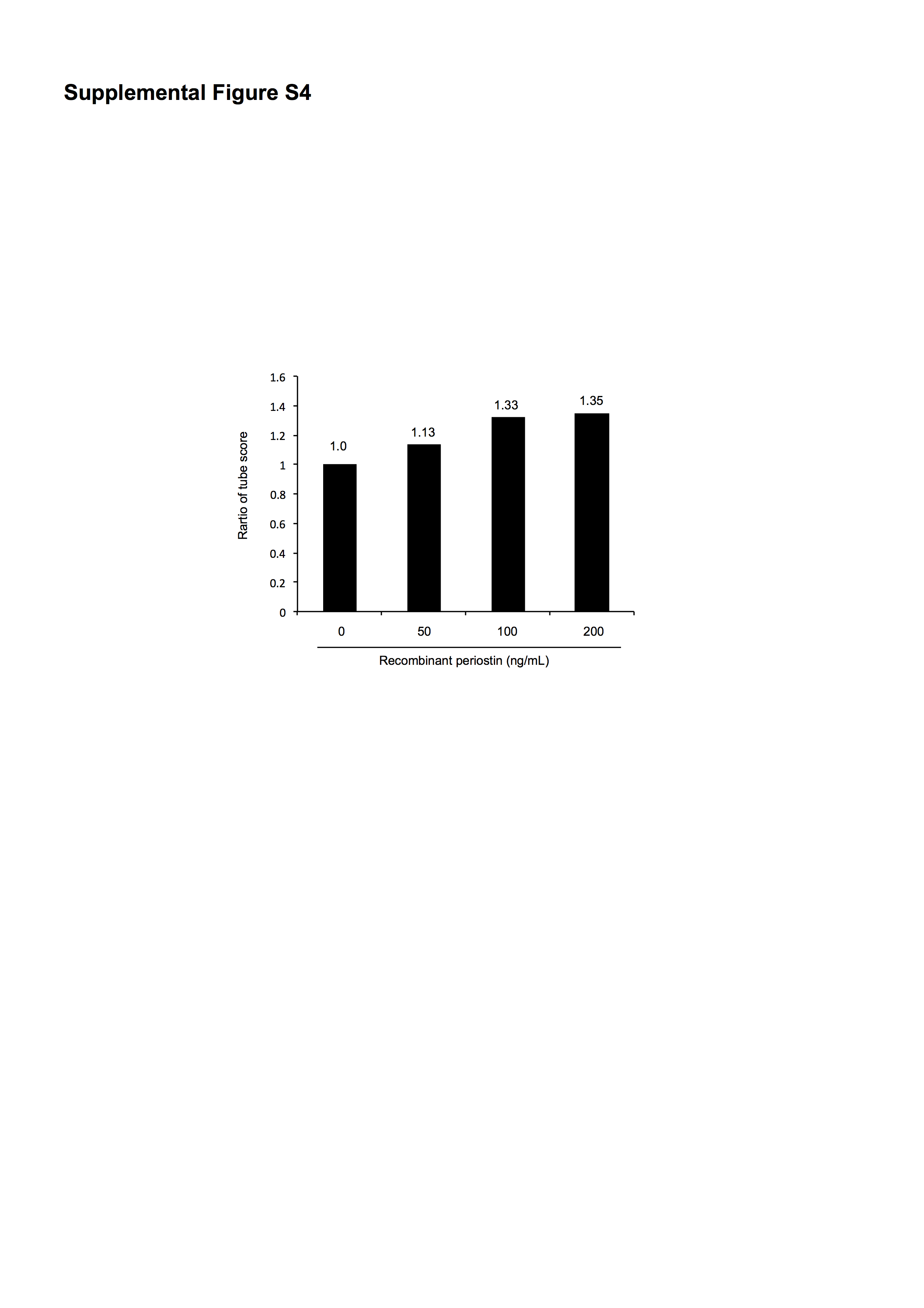

Supplement: Figure S4 — Direct involvement of periostin in tube formation of lymphatic endothelial cells. TR-LE cells were seeded onto matrigel-coated wells in the presence of periostin (0, 50, 100 or 200 ng/mL). The graph shows the tube score ratios after treatment with periostin for 9 h. The tube score of control was defined as 1.0. (TIFF) [file pone.0044488.s004.tiff]

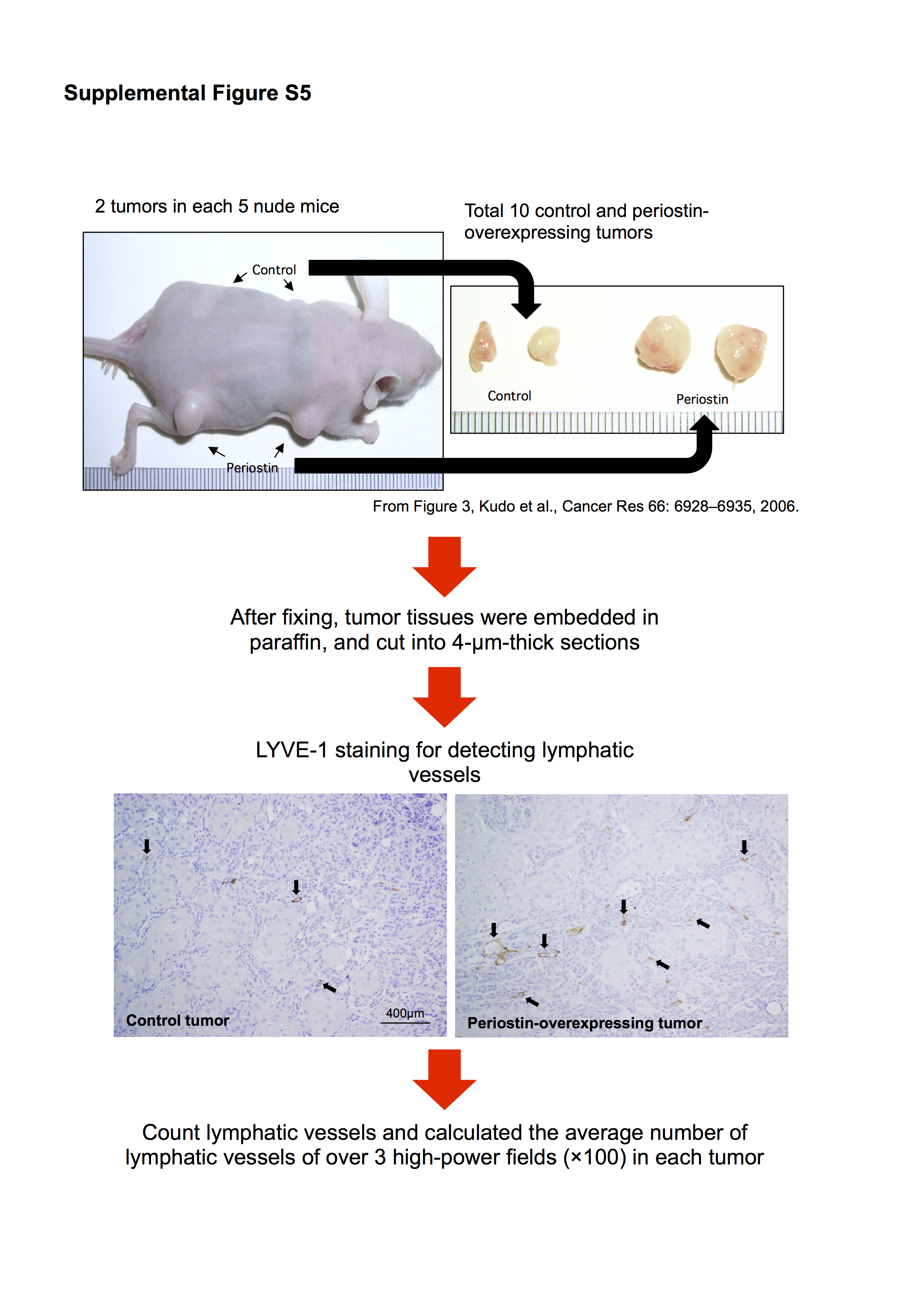

Supplement: Figure S5 — Number of lymphatic vessels in xenograft periostin-overexpressing tumors. Schema shows the method to count the number of lymphatic vessels in control and periostin-overexpressing tumors in xenograft model. (TIFF) [file pone.0044488.s005.tiff]

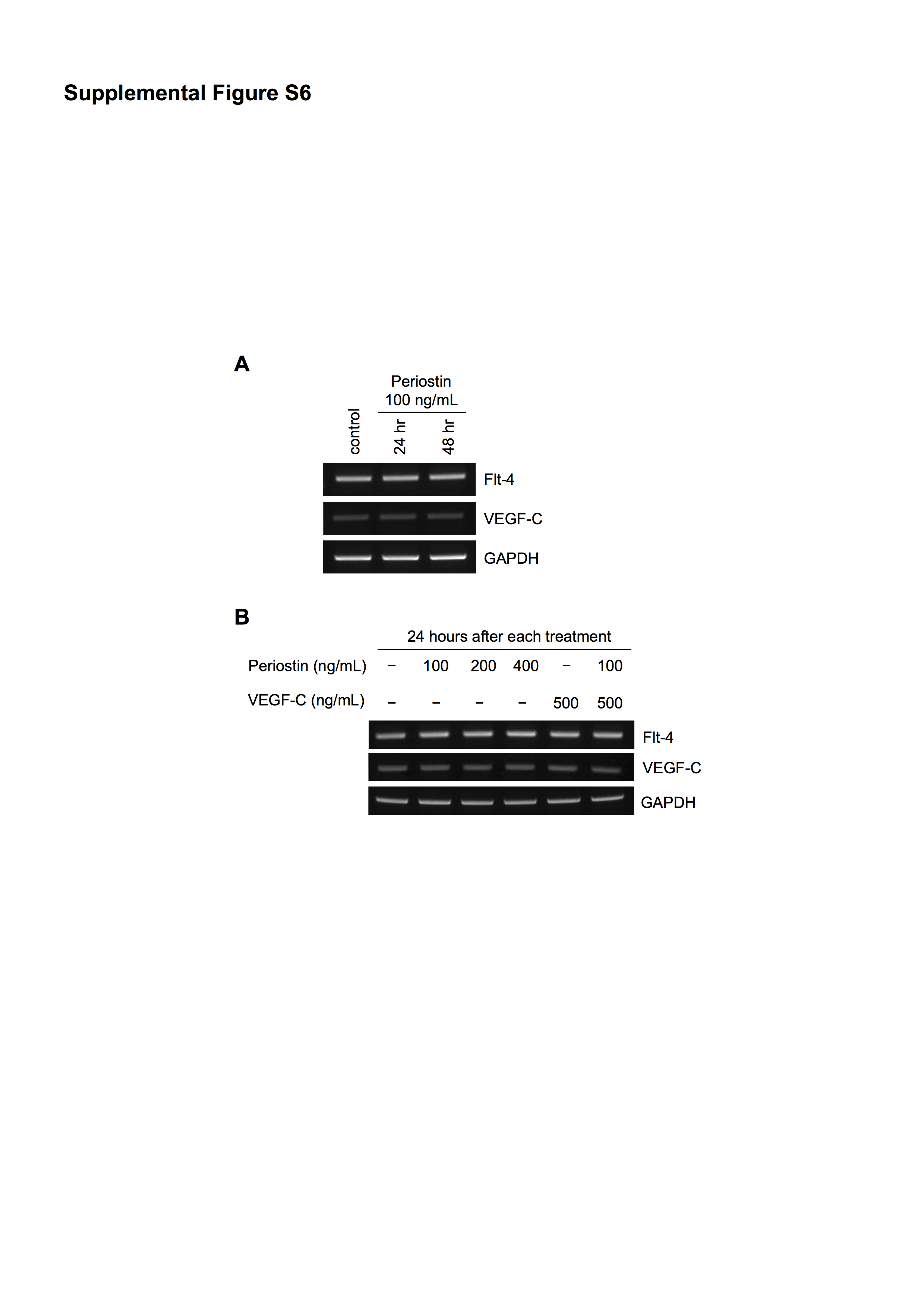

Supplement: Figure S6 — Periostin did not affect to Flt-4 expression. A: Expression of Flt-4 and VEGF-C mRNA was examined by RT-PCR in TR-LE cells after 24 and 48 hr of Periostin treatment (100 ng/mL). GAPDH expression was used as a loading control. Two pairs of primer sequences were; rat Flt-4, 5′-taaccgacctcctggtgaac-3′ (forward) and 5′-tgcacacactgcacaggtaa-3′ (reverse) (product size, 204 bp); rat VEGF-C, 5′-agcagccacaaacaccttct-3′ (forward) and 5′-ttagctgcctgacactgtgg-3′ (reverse) (product size, 285 bp); rat GAPDH, 5′-accacagtccatgccatcac-3′ (forward) and 5′-tccaccaccctgttgctgta-3′ (reverse) (product size, 452 bp). B: Expression of Flt-4 and VEGF-C mRNA was examined by RT-PCR in TR-LE cells after 24 hr of Periostin treatment (100, 200 and 400 ng/mL), VEGF-C treatment (500 ng/mL) and Periostin (100 ng/mL) and VEGF-C (500 ng/mL) treatment. (TIFF) [file pone.0044488.s006.tiff]

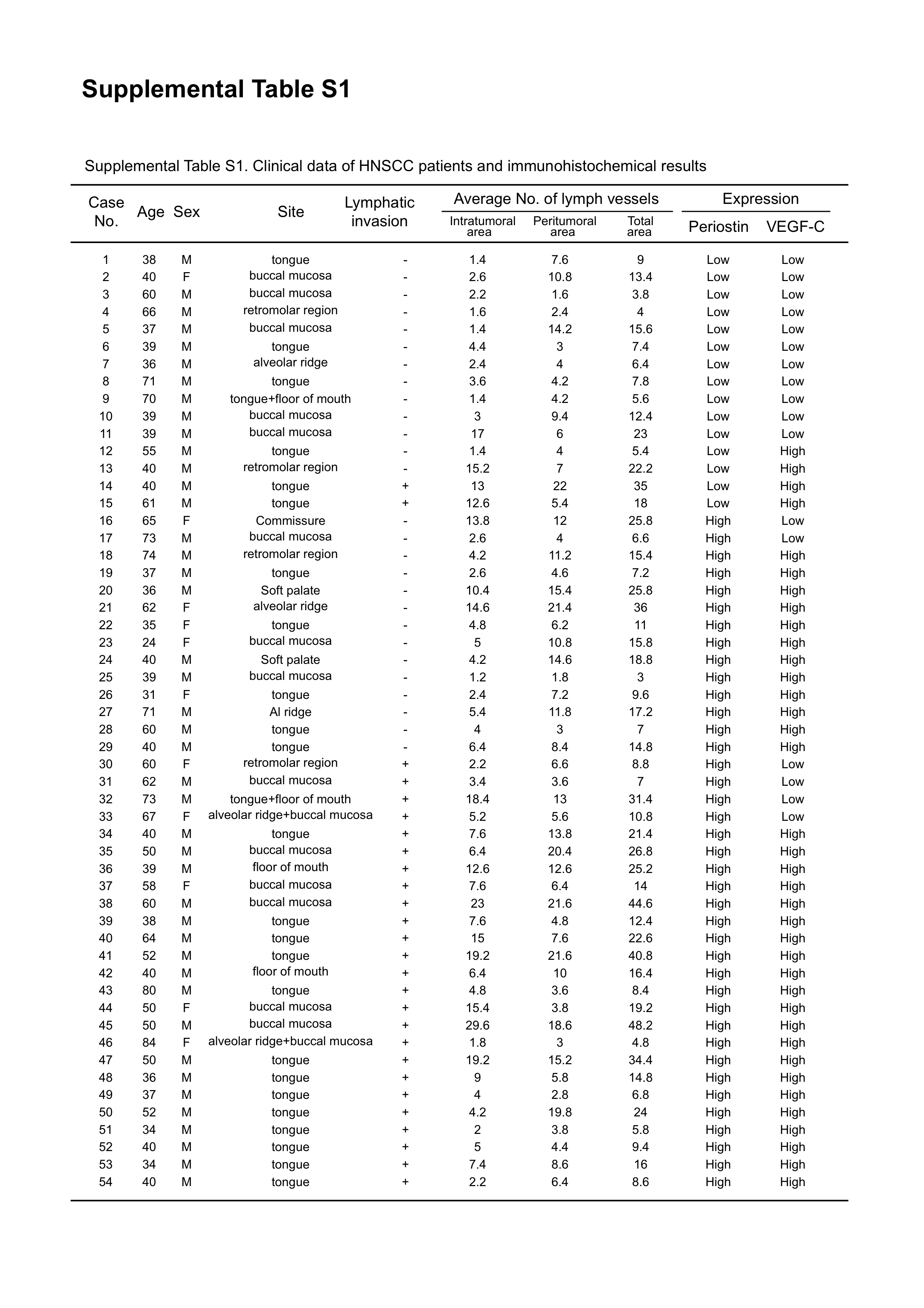

Supplement: Table S1 — Clinical data of HNSCC patients and immunohistochemical results. (TIFF) [file pone.0044488.s007.tiff]

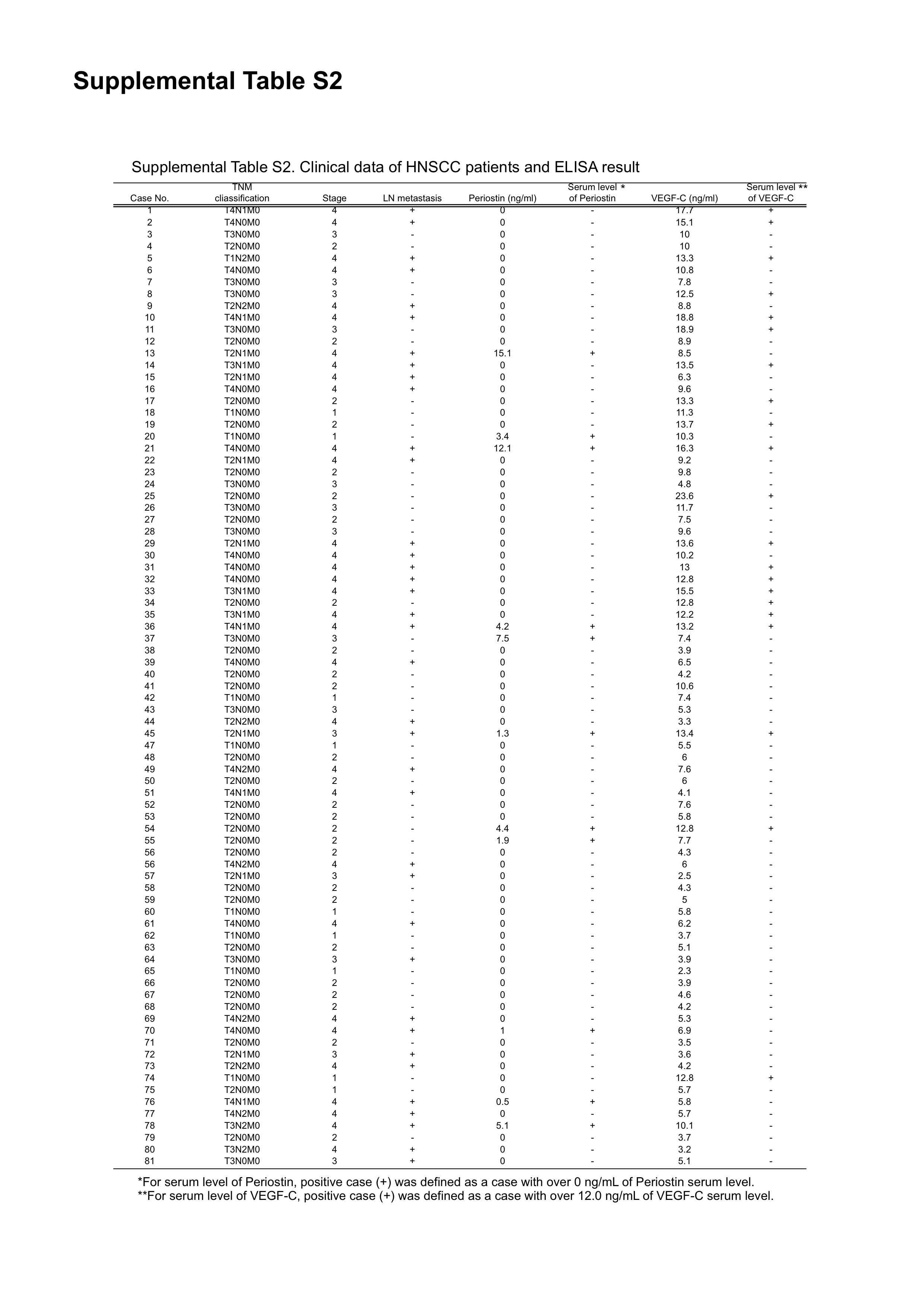

Supplement: Table S2 — Clinical data of HNSCC patients and ELISA result. (TIFF) [file pone.0044488.s008.tiff]
